# A meta-analysis of preventive psychosocial interventions against depressive and anxiety symptoms in older adults

**DOI:** 10.1017/S0033291726104607

**Published:** 2026-05-14

**Authors:** Sandra Saldivia, Joseph Aslan, Anabel Castillo-Carreño, Eleni Petkari, Jakob Pietschnig

**Affiliations:** 1 Universidad de Concepción, Chile; 2 CIADES – Center of Research and Action in Social Determination and Mental Health, Chile; 3 Faculty of Psychology and Humanities, Psychology Degree Program, Universidad San Sebastián, Concepción, Chile; 4 School of Psychology and Speech Therapy, Universidad de Malaga, Spain; 5 University of Vienna: Universitat Wien, Austria

**Keywords:** anxiety, depression, elderly, multiverse meta-analysis, preventive intervention, psychosocial interventions, RCT

## Abstract

Common mental disorders (CMDs) such as depression and anxiety are highly prevalent among older adults. While psychosocial interventions are increasingly recognized for their preventive potential, a comprehensive synthesis of their effectiveness with nonclinical elderly populations is pending. This study aimed to evaluate the effectiveness of such interventions in reducing depressive and anxiety symptoms among older adults with subclinical symptom levels and to examine potential moderators (that is, intervention type, length, delivery modality, and control group characteristics). A meta-analysis was conducted of 58 randomized controlled trials (RCTs) testing psychosocial interventions aimed at preventing depression and/or anxiety, using validated measures and targeting adults aged ≥60. Moderator variable effects were assessed through mixed-effects meta-regressions, and effect generality was examined using multiverse analyses. Psychosocial interventions showed a moderate postintervention effect in reducing depressive symptoms (*d* = −0.474) that remained nontrivial and modest at follow-up (*d* = −0.386) compared to control. For anxiety, a small-to-moderate effect was observed postintervention (*d* = −0.333), with a small, albeit nominally nonsignificant, effect at follow-up (*d* = −0.205) compared to control. No significant differences were found between intervention types or control conditions. Younger participants experienced greater reductions in depressive symptoms from pre-to-post-intervention and at follow-up, and in anxiety symptoms from pre-to-post-intervention only. Multiverse analyses showed that intervention effects generalized across numerous variables, thus indicating a remarkable robustness of the findings. Our findings demonstrate that it is important to implement psychosocial interventions in community settings, regardless of intervention type, to protect the elderly against CMDs.

## Introduction

Depression and anxiety – referred to as common mental disorders (CMDs) – are the most prevalent mental health conditions among older adults (Curran, Rosato, Ferry, & Leavey, 2020; Jalali et al., 2024), with a significant impact on health (Chen, Lee, Su, Mullan, & Chiu, 2017). CMDs are associated with greater functional impairment and physical illness, slower recovery from medical conditions, elevated suicide risk, and more frequent and longer hospitalizations (Berk et al., 2023; Webb & Chen, 2022).

First-line treatments for CMDs include pharmacotherapy and psychotherapy, with cognitive behavioral therapy (CBT) being the most common. Problem-solving strategies are also particularly effective with older adults (Simon, Moise, & Mohr, 2024). Adding mindfulness-based approaches has also shown significant benefits for older adults with comorbid anxiety and depression (Wang et al., 2024; Wuthrich et al., 2024). Strikingly, the prevalence of CMDs has not significantly decreased over the years, despite the increased availability of treatment options (Jorm, Patten, Brugha, & Mojtabai, 2017).

For this reason, preventive interventions may offer a valuable approach for protecting against CMDs, especially in older adults (Cuijpers, Van Straten, & Smit, 2005; Singh, Kumar, & Gupta, 2022). Evidence supports the efficacy of such interventions in treating preclinical levels of CMDs in older adults (de Oliveira, Dornelles, Gosmann, & Camozzato, 2023; Dworschak, Heim, & Maercker, 2022; Scazufca et al., 2024). However, the exact intervention effectiveness has not been determined. Previous meta-analyses have assessed the efficacy of psychosocial interventions (Forsman, Schierenbeck, & Wahlbeck, 2011), CBT (Vedel, Larsen, & Aamand, 2020; Wang et al., 2024; Wuthrich et al., 2021; Wuthrich et al., 2024), educational programs (Conejo-Cerón et al., 2017), mind–body interventions such as yoga (Dong et al., 2024), and physical activity interventions (Ahmad, Syam, & Saleh, 2023; Podolski et al., 2023) in reducing CMD symptoms in older adults. However, such endeavors present several limitations, namely, they (1) focus on specific interventions, (2) often mix community-dwelling samples with those diagnosed with clinical depression/anxiety; (3) do not account for potential interactions between moderating variables, and (4) mix RCT findings with noncontrolled trials.

Thus, this meta-analysis aims to define the effects of preventive psychosocial interventions for older adults with nonclinical levels of CMD symptoms. Secondary objectives include identifying factors that may influence the effectiveness of these interventions, such as intervention type (e.g., mind–body, psychology-based, multi-component), length, delivery modality (group versus individual), and comparison group type (e.g., treatment as usual (TAU), waiting list/no treatment, active comparator, nonspecific factor component control).

## Methods

This meta-analysis was registered at PROSPERO (CRD463398) and prepared according to the PRISMA guidelines (Page et al., 2021; see Supplementary File 1).

### Literature search

A systematic search was performed in PubMed/MEDLINE, PsycINFO, Web of Science, Scopus, and The Cochrane Library. The search ran until December 2024, comprising search terms related to the population (elderly), intervention type (preventive interventions against anxiety/depression), and study design (RCT) and was based on abstract, title, and keywords. Hits were restricted in publications in English and Spanish. The search string and strategies can be seen in Supplementary File 2.

### Inclusion and exclusion criteria

To be eligible, primary studies needed to (i) follow an RCT design, (ii) study older adults (mean age > 60 years old), (iii) include a valid instrument measuring depression and/or anxiety, (iv) focus on people attending any outpatient mental health or community setting; (v) test the effectiveness of a psychosocial intervention (that is, mind–body-based; psychology-based; multicomponent; other) delivered with human component (that is clinician, trainer), (vi) target, or measure as an outcome, depression and/or anxiety through any standardized instrument, (vii) include a control group of users receiving TAU, being on the waiting list/no treatment, receiving a specific/active control, or a nonspecific control; and (viii) report sufficient statistical parameters to allow effect size calculation or provide the data upon request. The following were excluded: (i) non-RCTs, publications on the same data, and insufficient reporting; (ii) non-English/Spanish publications; (iii) studies applying pharmacological, treatment, or recovery programs; (iv) studies applying interventions not including a human provider (that is, apps, self-help); and (v) samples with diagnosed mental disorders (including major depression/anxiety), substance abuse, or dementia.

### Screening and data extraction

Three researchers (JA, AC, and EP) performed the database searches; two of them (JA and AC) removed duplicates. Two of them (JA and AC) screened the search output independently based on titles/abstracts and accessed and screened the potentially eligible full-texts. Discrepancies were discussed with two reviewers (SS and EP). Extracted data are available at https://osf.io/e7vxz/. In all, 21 corresponding authors were contacted, and 14 provided missing information for eligible studies (66%).

The following data were coded: title, author, publication date, location, language; study design: intervention duration, follow-up periods; participant demographics: age, sex; sample size for intervention and control group; intervention characteristics: type (mind–body based, psychology-based, multicomponent, other), duration (in weeks), modality (group/individual); control group intervention/condition (TAU, waiting list/no treatment, active comparator/specific factor component, non-specific factor component); outcomes: depressive/anxiety symptoms; statistical parameters: means, standard deviations for intervention and control groups.

Interventions were grouped into four categories based on their content as described in the primary studies:

1. Mind–body-based: including physical exercise activities (i.e., Tai-Chi, aerobic and resistance training, Xbox Kinect exercise training, home-based exergame program, walking, yoga).
2. Psychology-based: based on a theory-driven psychological approach (i.e., CBT, behavioral activation, cognitive training, mindfulness, life story review, psychoeducation).
3. Multicomponent: combining components of several approaches (i.e., social activities with psychoeducation; psychoeducation with mindfulness; exercises and diet; exercises and social activities; physical, cognitive, and social activities).
4. Other: interventions not fitting suitably in the previous categories (i.e., pet support, attending sports activities, photograph appreciation).

Control group conditions were grouped into four groups based on the Gold et al. (2017) approach adjusted to the eligible studies content:

1. TAU: care as usual.
2. Waitlist control/no treatment: no treatment during the intervention period – could be offered after the trial.
3. Active control: evidence-based treatment different from the experimental condition; may include structured activities or techniques that are considered therapeutic.
4. Nonspecific factor component control: structured programs with the same duration and frequency as the experimental condition but not including activities or techniques considered therapeutic.

### Outcome and quality assessment

The primary outcome was the depression and/or anxiety symptom change. Symptoms were measured through standardized instruments (e.g., Geriatric Depression Scale-GDS/Patient Health Questionnaire-PHQ for depression; Geriatric Anxiety Inventory-GAI/Hospital Anxiety Depression Scale-HADSHADS for anxiety). Two reviewers (JA and AC) assessed the study quality using the Effective Public Health Practice Project Quality Assessment Tool (EPHPP; Thomas, Ciliska, Dobbins, and Micucci (2004)). Studies were rated based on study design, blinding, confounders, instruments, psychometric characteristics, and withdrawals/dropouts. The GRADE system was applied to assess certainty of evidence for depressive and anxiety symptoms, based on study design, risk of bias, inconsistency, imprecision, and publication bias (Atkins et al., 2004).

### Statistical analyses

We calculated the summary effects of standardized mean differences (Cohens *d*) using the means of random effect calculations following current recommendations (Borenstein, Hedges, Higgins, & Rothstein, 2021). Heterogeneity was assessed by calculating *I*-squared values, which we interpreted in terms of well-established thresholds, with values from 0 to 25% indicating no, 25 to 50% indicating little, 50 to 75% indicating moderate and 75 to 100% indicating strong between-studies heterogeneity (Higgins, Thompson, Deeks, & Altman, 2003).

We used single precision-weighted regression models to assess the effects of intervention type (a subgroup summary effect was calculated for each distinct intervention, i.e., mind–body-based, psychology-based, multicomponent program, other), modality (group versus Individual versus mixed), control group interventions (TAU, being in waiting list or no intervention, specific or nonspecific intervention), cognitive function change, age, sex, outcome being primary or secondary, publication year, and study quality. For categorical variables, categories were dummy-coded to allow comparisons. Effect sizes were interpreted based on Cohen’s (2013) well-established thresholds (i.e., *d*s = 0.2, 0.5, and 0.8 represent the lower thresholds of small, moderate, and large effects, respectively). Analyses were performed in the open-source software environment R 4.2.0 (R Core Team, 2022) by means of the package metafor (Viechtbauer, 2010).

### Dissemination bias

We visually inspected funnel plots for asymmetry indications, and we used four formal publication bias-detection methods. First, we used trim and fill analyses that allow a formal assessment of funnel plot asymmetry by iteratively trimming excess studies on one side of an asymmetric funnel and re-estimating the summary effect until funnel plot symmetry is observed. Subsequently, trimmed studies are re-entered into the funnel and studies with equivalent weights and effect sizes are added on the opposite side of the funnel. This method can be interpreted in terms of a sensitivity analysis (Duval & Tweedie, 2000). Second, we used the Begg and Mazumdar (1994) rank correlation method that assesses correlations between effect sizes and their sampling variances, where significant associations are indicative of confounding bias. Third, we calculated Sterne and Egger’s regression method (Sterne & Egger, 2005) in which effect sizes are regressed on their precision estimates, with significant results indicating bias. Finally, we assessed evidence for possible excess significance of the number of observed primary study effects (Ioannidis & Trikalinos, 2007). We interpreted all results according to current standards as outlined by Siegel, Eder, Wicherts, and Pietschnig (2022).

### Multiverse analyses

Different (reasonable) choices of researchers in approaching the design of the studies and the data analysis (i.e., the so-called researcher degrees of freedom) have been shown to have, in many cases, considerable impact on the outcome of the study (Simonsohn, Simmons, & Nelson, 2020) In the meta-analytical context, these choices pertain typically to the questions about which data to analyze (the so-called ‘which factors’) and which analytical methods to use (the so-called ‘how factors’; (Voracek, Kossmeier, & Tran, 2019). Some effects may only be observable under very specific circumstances or, in the worst case, may even represent a spurious finding that is only observable due to chance in a certain dataset after specific choices have been made, whereas other effects show a large generality by manifesting themselves regardless of the choices that have been made. Specification curve analysis and combinatorial meta-analyses are useful to show the meaningfulness, generality, and specificity of effects under scrutiny.

### Specification curve

In specification curve analyses, one aims to assess all possible summary effects that can be calculated based on reasonable analytical choices that can be made in subsetting (‘which factors’) and analyzing (‘how factors’) the available data. We presently account for five which factors, namely control group type (nonspecific versus specific versus TAU, versus waitlist versus either control type), intervention type (multicomponent versus psychology-based versus mind–body-based versus other versus either intervention), quality assessment (weak versus moderate versus strong versus any), primary outcome (no versus yes versus either), treatment setting (group versus individual versus mixed versus any) and one how factor, namely, the calculation method (REML: random effects maximum likelihood versus RE-DL: random effects DerSimonian-Laird versus FE: fixed effect). This resulted in 5*5*3*4*4*3 = 3,600 reasonable ways (i.e., specifications) to calculate a summary effect in our present meta-analysis. However, we considered only those specifications that included at least two primary studies for summary effect calculations, thus reducing the number of outcomes accordingly.

### Combinatorial meta-analysis

It can be highlighted that the specification curve falls short of assessing the generality and salience of a given effect, because not all factors that can influence the manifestation of a summary effect may necessarily be known. To account for this possibility, the combinatorial meta-analytical approach has been introduced (Olkin, Dahabreh, & Trikalinos, 2012. By means of this method, not only the reasonable but all 2^*k*-1^ possible combinations of the available studies are considered to yield potentially meaningful representations of the scrutinized effect. In our analyses, this approach yields 2^59^ = 576,460,752,303,423,488 and 2^23^ = 8,388,608 combinations for pre-to-post as well as pre-to-follow-up comparisons, respectively, in depression and 2^29^ = 536,870,912 and 2^18^ = 262,144 combinations, respectively, in anxiety. As these numbers of effect estimations exceed the computational power of most standard equipment, we drew a random sample of 100,000 combinations within each of these calculations. This approach allowed us to inspect the potential systematic patterns in terms of the relation of summary effect sizes with their associated between-studies heterogeneity by means of GOSH plots (Graphic Display of Heterogeneity; (Olkin et al., 2012).

## Results

### Final sample

**Figure 1.**
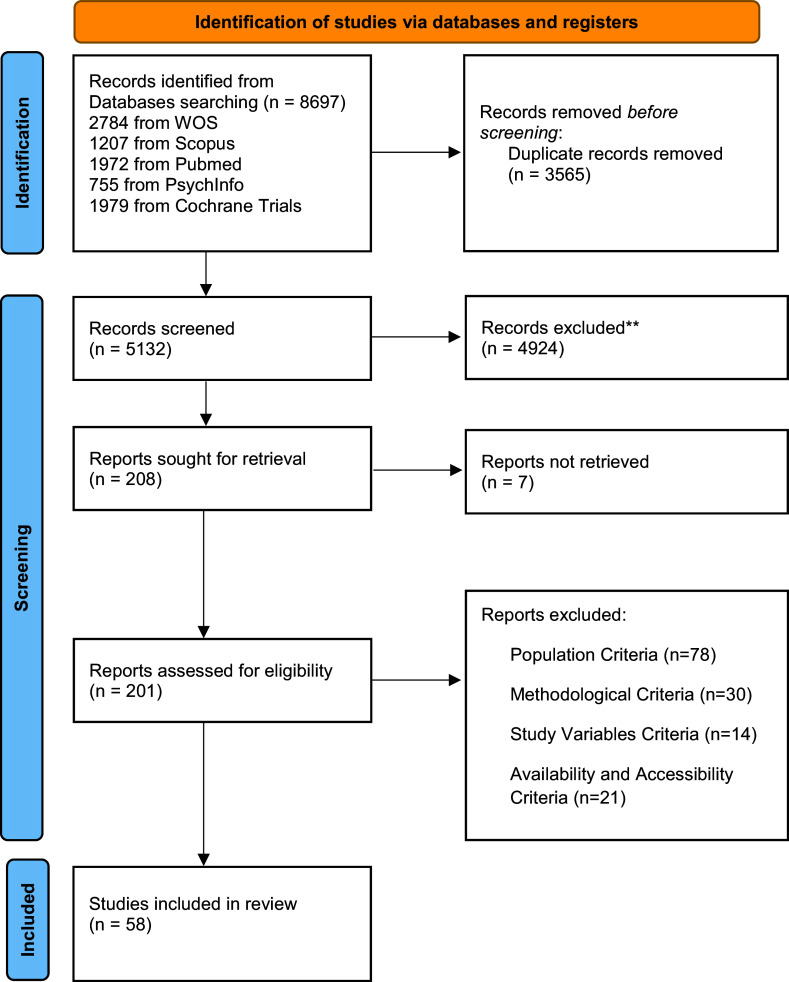
PRISMA Flowchart *Source:* Page et al., 2021. This work is licensed under CC BY 4.0. To view a copy of this license, visit https://creativecommons.org/licenses/by/4.0/.

The inclusion criteria were met by 58 independent studies (56 on depression and 26 on anxiety, i.e., almost half of the studies including both outcomes), reporting *k* = 60 effect sizes for depression and *k* = 30 effect sizes for anxiety comparing pre-to-post intervention levels. Also, *k* = 24 effect sizes were reported for depression and *k* = 19 effect sizes for anxiety comparing pre-intervention to follow-up (Check Figure 1 for the PRISMA flowchart for study inclusion; References are displayed in Supplementary File 14.).

For studies focusing on depression, there were 5,517 participants, whereas for anxiety there were 2,999 participants. Of those, there were 2,449 participants with available data on follow-ups for depression and 2,080 for anxiety.

The mean participant age for studies focusing on depression was *M* = 72.07 (*SD* = 4.27) and for anxiety, it was *M* = 71.40 (*SD* = 4.07). For studies focusing on depression, 70.14% were female, whereas for anxiety, the percentage was 68.02%.

There were 27 studies from the United States, Canada, Australia, and Europe, 23 studies from South Asia, and 4 studies from South America and the Middle East.

### Interventions and controls description

Out of the 56 studies comparing the effects of an intervention on depression levels, 34 studies use psychology-based interventions, 12 use mind–body-based interventions, five use multicomponent therapy, and five use other therapies.

In terms of comparisons, 21 studies compared the interventions’ effectiveness with TAU, 16 studies with waitlist, 12 studies with specific comparators, and 7 studies with nonspecific controls. Studies reporting follow-ups would most commonly follow up patients for 52 weeks, with the longest follow-up being 192 weeks.

When it comes to anxiety, out of 26 studies, 19 applied psychology-based interventions, t3 applied mind–body-based interventions, 3 multicomponent interventions, and 1 applied other intervention. There were 12 studies comparing the control groups with TAU, 5 with waitlist, 6 with specific comparator, and 2 with the nonspecific comparator. The longest follow-up time was 192 weeks. (See Table 1 for study characteristics.)

**Table 1.** Characteristics of included studies

Authors, year, country	Sample, mean age (SD), % female (Intervention group/Control group))	Primary intervention type	Intervention group: description	Control group type	Control group: description	Intervention duration and type (group/individual)	Follow-up	Instruments for depression/anxiety symptoms and cognitive function	Assessment time-points	Main conclusions
Alegria et al., 2019, USA	Positive Minds-Strong Bodies N = 153 Age M = 74.9 (7.1) 80.4% female TAU N = 154 Age M = 75.4 (8.0) 81.2% female	Multicomponent	Positive Minds-Strong Bodies (PMSB): psychoeducation, mindfulness, cognitive restructuring, noticing and overcoming unhelpful thoughts, and creating a self-care plan, exercise training.	Treatment as usual	Enhanced usual care. A call by staff every two weeks to check mental health status. Empathetic support. Booklet about caring for one’s mental and physical health.	24w Mixed	52w	Anxiety Generalized Anxiety Disorder 7-item Scale (GAD–7)	Pre, Post (36w), Follow up (52w)	The intervention was effective for improving mood but not anxiety. The findings were maintained at follow-up.
Almeida et al., 2020, Australia	Behavioral Activation phone N = 154 Age M = N/A 51.3% female TAU N = 153 Age M = N/A 51% female	Psychology-Based	Behavioral Activation phone delivered program booklet with information on depression; symptom recognition and management, keeping a diary, action plan, activity scheduling plus usual care.	Treatment as usual	Usual care support from the staff and feedback about symptoms after assessment.	8w Individual	8w	Depression Patient Health Questionnaire (PHQ–9) Anxiety Generalized Anxiety Disorder 7-item Scale (GAD–7)	Pre, 26w, Follow up (52w)	The intervention significantly reduced depression and anxiety symptoms over one year, with a statistically significant difference in GAD–7 scores at 26 weeks.
Ayudhaya et al., 2020, Thailand	Behavioral Activation N = 41 Age M = 70.54 (5.39) 80.49% female TAU N = 41 Age M = 69.20 (7.06) 78.05% female	Psychology-Based	Behavioral Activation Psychoeducation, activity monitoring, activity scheduling, and modification	Treatment as usual	Usual care, regular physical examinations, review of current health symptoms upon the individual’s health needs, and psychoeducation.	12w Group	39w	Depression Thai geriatric depression scale (TGDS) Anxiety-Stress Depression Anxiety Stress Scales (DASS)	Pre, Post (12w), Follow up (26w,39w)	Depression and stress improved significantly in the Behavioral Activation group compared to the usual care-only group, whereas anxiety score improved significantly only at 26 weeks.
Bae et al., 2019, Japan	Multicomponent N = 41 Age M = 75.5 (6.0) 43.9% female Health Education N = 42 Age M = 76.4 (5.1) 52.4% female	Multicomponent	Multicomponent Physical activities, cognitive activities and social activities.	Non-specific factor component control	Health education: classes on oral care (e.g. dental care and swallowing exercise) and nutrition (e.g. risk factor for malnutrition, health effects, and prevention).	24w Group	–	Depression 15-item Geriatric Depression Scale (GDS–15) Cognitive Function Mini-Mental State examination (MMSE)	Pre, Post (24w)	There were no significant differences between the intervention and the control group in depression levels
Boen et al., 2012, Norway	Intervention N = 77 Age M = N/A 59.5% female TAU N = 55 Age M = N/A 54.7% female	Multicomponent	Physical training Self-help discussing topics around safety, social relations, and aging.	Treatment as usual	Usual care daily activities.	52w Group	–	Depression Beck Depression Inventory (BDI)	Pre, Post (52w)	No differences between the control and the intervention group
Brenes et al., 2007, USA	Exercise N = 14 Age M = 73.5 (7.8) 64% female TAU N = 12 Age M = 73.9 (5.8) 50% female	Mind–Body-Based	Exercise, aerobic and resistance training.	Treatment as usual	Usual care discussion on the participants’ health status.	16w Group	–	Depression 15-item Geriatric Depression Scale (GDS–15)	Pre, Post (16w)	All participants had a decline in depressive symptoms. There was a trend of exercise being superior to usual care, though not significant.
Catalayud et al., 2021, Spain	Cognitive Stimulation N = 101 Age M = 72.08 (5.373) years 68.3% female TAU N = 100 Age M = 73.75 (5.899) years 63% female	Psychology- based	Cognitive Stimulation: Exercises on memory, orientation, language, calculation, perception, etc.	Treatment as usual	Usual care.	10w Group	52w	Depression 15-item Geriatric Depression Scale (GDS–15) Anxiety Goldberg Anxiety Scale Abbreviated Cognitive Function Mini-examen Cognoscitivo (MEC-MMSE)	Pre, Post (10w), Follow up (26w, 52w)	The results showed significant improvements in the Mini Mental State Examination scores in the intervention group, regardless of gender, age, or educational level. No significant differences were found in anxiety or depression levels.
Casemiro et al., 2018, Brazil	Health Education Group N = 10 Age M = 68 (6.05) 70% female TAU N = 12 Age M = 77.3 (6.31) 75% female	Psychology-Based	The psychoeducation intervention involved weekly sessions on 10 topics, starting with 20-minute group discussions and followed by researcher-led presentations. Pre-session group dynamic activities were conducted to enhance engagement and understanding.	Treatment as usual	Every two weeks, they received a phone call. The purpose of these calls was to ask subjects about their health status, routine, and to learn about the start of any new activities.	20w Group	–	Depression Beck Depression Inventory (BDI) Anxiety Beck Anxiety inventory (BAI) Cognitive Function Mini-Mental State Examination (MMSE)	Pre, Post (20w)	A health education intervention using active strategies and group dynamics improved cognitive functions, particularly memory, language, and orientation, but showed no statistically significant impact on depressive or anxiety symptoms despite participant feedback.
Cavalcante et al., 2021, Brazil	Resistance Exercise with instability N = 22 Age M = 71 (6.0) 77% female Resistance Exercise N = 23 Age M = 71 (6.0) 78% female Health Education N = 22 Age M = 71 (4.0) 77% female	Mind–Body-Based	Resistance Exercise with Instability (REI): Seven exercises for three sets and repetitions, abdominal exercises plus instability devices. Resistance Exercise (RE): Seven exercises for three sets and repetitions, abdominal exercises.	Active comparator	Health Education weekly health education seminars including lectures on prevention and treatment of health-related issues, maintenance of healthy behaviors (e.g., physical activity), or stretching and relaxation classes.	12w Group	–	Depression 15-item Geriatric Depression Scale (GDS)	Pre, Post (12w)	Compared with the control group, no significant between group differences were observed for the depression scores after trial completion in both Resistance Exercise with Instability and traditional Resistance Exercise.
Chan and Lo, 2023, China	Zentangle N = 23 Age M = 72.9 (5.6) 87% female Waitlist N = 23 Age M = 73.7 (7.35) 91.3% female	Psychology-Based	Zentangle Mindfulness-based: Cultivate self-compassion and promote calmness through learning different drawing techniques and patterns.	Waitlist/No-treatment control	Waitlist	6w Group	12w	Depression Patient Health Questionnaire (PHQ–9)	Pre, Post (6w), Follow up (12w)	The Zentangle method effectively reduced depressive symptoms and increased self-compassion in older adults, with improvements sustained six weeks post-intervention, making it a valuable supplementary approach for late-life depression.
Chan et al., 2013, Singapore	Life story review N = 14 Age M = 70.4 (6.8) 85.7% female Control Group N = 14 Age M = 68.9 (7.1) 75% female	Psychology-Based	Life story review: memories of his/her childhood, adolescence, adulthood years as well as his views on his/her current life situation.	Nonspecific factor component control	No intervention	8w Individual	–	Depression 15-item Geriatric Depression Scale (GDS–15)	Pre, Post (8w)	Significant reductions in depression scores were found in the intervention group compared to the control group.
Cieślik et al., 2023, Poland	Immersive virtual reality N = 30 Age M = 68.77 (5.57) 100% female Control Group N = 30 Age M = 67.53 (5.51) 100% female	Psychology-Based	Immersive virtual reality Japanese garden aesthetics, relaxation, and elements of Ericksons Psychotherapy Plus general fitness low-intensity general fitness training sessions	Active comparator	General fitness training Plus relaxation and psychoeducation	4w Group	–	Depression 30-item Geriatric Depression Scale (GDS–30) Anxiety/Depression Hospital Anxiety and Depression Scale (HADS)	Pre, Post (4w)	Statistically significant differences were observed between the groups in the post-treatment assessment however, these differences were significantly higher in the immersive virtual reality group.
De Lima et al., 2021, Brazil	Exercise N = 15 Age M = 67.2 (4.4) 86.6% female Control Group N = 14 Age M = 68 (6.1) 71.4% female	Mind–Body-Based	The Xbox Kinect training featured six activities targeting balance, flexibility, strength, and coordination, including yoga-like stretches, boxing, soccer, ball lifting, and block breaking, combining flexibility, strength, and coordination exercises.	Waitlist/No-treatment control	No activity	6w Group	–	Anxiety Spielberger State–Trait Anxiety Inventory (STAI)	Pre, Post (6w)	No significant differences in state anxiety between the two groups.
Dear et al., 2014, Australia	Cognitive Behavioral Therapy N = 33 Age 65.39 (4.68) 67% female Waitlist N = 37 Age M = 65.51 (5.81) 54% female	Psychology-Based	Cognitive Behavioral Therapy (Managing Stress and Anxiety Course)	Waitlist/No-treatment control	Waitlist	8w Individual	–	Depression Patient Health Questionnaire (PHQ–9) Anxiety Generalized Anxiety Disorder 7-item Scale (GAD–7)	Pre, Post (8w)	The GAD–7 and the PHQ–9 scores of the treatment group at posttreatment, were all significantly lower than their pretreatment scores.
Delholm et al., 2022, Spain	Emotional Intelligence N = 60 Age M = 68.59 (6.39) 82.4% female Control Group N = 65 Age M = 67.45 (6.43) 78.3% female	Psychology-Based	The Emotional Intelligence-based intervention included exercises on emotional reflection, introspection, self-awareness, emotion–cognition interaction, empathy, assertiveness, stress management, coping strategies, and emotional regulation.	Waitlist/No-treatment control	They were not exposed to the intervention.	10w Group	–	Depression Centre for Epidemiologic Studies-Depression Scale (CES-D)	Pre, Post (10w)	The intervention led to a significant reduction in depressive symptoms and hopelessness among the participants.
Denkova et al., 2024, USA	Intervention group N = 26 Age M = 72.15 (5.97) 88.46% female Control group N = 27 Age M = 70.67 (5.77) 85.19% female	Psychology-based	Mindfulness training program. Course delivered online via Zoom in 2-h trainer-led sessions that occurred once a week. Participants met as a group with opportunities to interact with each other and the instructor.	Waitlist/No-treatment control	Surveys	4w Group	–	Depression Patient Health Questionnaire-ultra brief 4-item form (PHQ–4)	Pre, Post (4w)	Mindfulness training (MT) improved some aspects of mindfulness and well-being but had no significant impact on psychological health or cognitive performance. Weekly changes in negative mood were observed, influenced by the evolving COVID–19 context.
Ghodsbin et al., 2015, Iran	Laughter therapy program N = 36 Age M = 68.55 (6.24) 72% female Control Group N = 36 Mean Age: 67.27 (5.87) 67% female	Psychology-based	Laughter therapy program incorporating breathing exercises, physical activities, and laughter techniques. For 6-week with two 90-minute sessions per week.	Waitlist/No-treatment control	No intervention	6w Group	–	Depression/Anxiety General Health Questionnaire (GHQ–28)	Pre, Post (6w)	Laughter therapy significantly improved general health and its subscales in elderly participants, with notable enhancements observed in the experimental group post-intervention.
Göks and Duru, 2021, Turkey	Progressive Muscle Relaxation N = 21 Age M = 69.2 (6.2) 100.0% female TAU N = 28 Age M = 71.0 (5.7) 100.0% female	Psychology-Based	The Progressive Muscle Relaxation (PMR) intervention involved 28-minute recorded exercises, applied three times a week for 8 weeks. Participants received weekly reminders, and those with irregular practice completed 24 sessions in total.	Treatment as usual	TAU	8w Individual	–	Depression 15-item Geriatric Depression Scale (GDS)	Pre, Post (8w)	The Intervention group got a statistically significant difference in the mean GDS–15 score of the women in the intervention group compared to the control group.
Gomez-Soria and Peralta-Marrupe, 2020, Spain	Cognitive Stimulation Program N = 54 Age M = 74.3 (5.8) 87.0% female TAU N = 68 Age M = 75.6 (6.2) 69.1% female	Psychology-Based	The Cognitive Stimulation Program consisted of 10 weekly 45-minute sessions over 10 weeks, including four parts: (a) reality orientation, (b) cognitive aspect explanation, (c) individual exercises, and (d) group correction.	Treatment as usual	TAU	10w Group	192w	Depression 15-item Geriatric Depression Scale (GDS–15) Anxiety Goldberg Anxiety Sub-Scale (Goldberg) Cognitive Function Mini-Mental State examination Spanish version (MEC–35)	Pre, Post (10w), Follow Up (24w, 52w, 192w)	The intervention group showed a significant improvement in cognitive function at both timepoints, post-test and 24 weeks follow-up. The intervention did not improve the performance of instrumental ADLs or depression or anxiety levels.
Gomez-Soria et al., 2023, Spain	Cognitive Stimulation Program (N = 160) Level deterioration group: N = 49 Age M = 74.16 (0.8) 38.9% female Moderation deterioration group: N = 10 Age M = 77.90 (1.75) 28.6% female No deterioration group: N = 50 Age M = 72.34 (0.8) 27.7% female Subtle cognitive impairment group: N = 50 Age M = 71.82 (0.72) 41% female TAU (N = 177) Level deterioration group: N = 59 Age M = 75.15 (0.78) 36.1% female Moderation deterioration group: N = 18 Age M = 82.39 (1.10) 50% female No deterioration group: N = 51 Age M = 71.69 (0.77) 27.7% female Subtle cognitive impairment group: N = 50 Age M = 75.9 (0.8) 35% female	Psychology-Based	The Cognitive Stimulation, program was personalized to participants’ cognitive levels and occupational variables. It involved 45-minute weekly sessions over 10 weeks, divided into four groups. Each session began with a theoretical explanation of the neuropsychological constructs addressed.	Treatment as usual	TAU	10w Group	52w	Depression 15-item Geriatric Depression Scale (GDS–15) Anxiety Goldberg Anxiety Sub-Scale (Goldberg) Cognitive Function Mini-Mental State examination Spanish version (MEC–35)	Pre, Post (10w), Follow up (24w, 52w)	The intervention showed a tendency of improvement on global cognition and different cognitive functions for groups with no deterioration or level deterioration. The group with moderate deterioration improved in anxiety.
Hae-Jin et al., 2016, Korea	Pet Insects N = 56 Age M = 71.1 (5.5) 77.1% female Heath Advice N = 53 Age M = 72.7 (5.7) 84.8% female	Other	The Pet Insects intervention provided participants with 5 crickets in a cage, fodder, and care instructions from an entomology expert. Weekly calls encouraged proper care and healthy lifestyle adherence, with replacement crickets provided if needed.	Non-specific factor component control	Heath Advice	8w Individual	–	Depression 15-item Geriatric Depression Scale (GDS–15) Anxiety Beck Anxiety inventory (BAI) Cognitive Function Mini-Mental State examination (MMSE)	Pre, Post (8w)	Caring for insects, which is cost-effective and safe, was associated with a small to medium positive effect on depression and cognitive function in community-dwelling elderly people.
Hardman et al., 2020, Australia	Exercise N = 24 Age M = 76.5 (7.4) 69.2% female Diet N = 25 Age M = 77.5 (7.4) 80.0% female Exercise + Diet N = 24 Age M = 77.7 (7.38) 70.8% female TAU N = 27 Age M = 78.2 (5.8) 66.7% female	Multicomponent	Exercise: Participants walked 30–40 minutes daily, starting at 15 minutes/day and increasing over a month, with progress tracked via a pedometer. Diet: Participants followed a 6-week Mediterranean meal plan with recipes incorporating extra-virgin olive oil as the primary fat source. Exercise + Diet: Participants combined both exercise and diet interventions	Treatment as usual	TAU	6w Individual	24w	Anxiety/Depression/ Stress Depression Anxiety Stress Scales (DASS)	Pre, Post (24w)	While there was no significant change in overall memory performance, there was a significant improvement in spatial working memory performance in the combined exercise and diet group, relative to controls. This combined intervention group also showed an overall improvement in their emotional state, as assessed by the DASS, as did the exercise-only group.
Hong and Lee, 2023, Korea	Comprehensive elderly care application N = 30 Age M = 71.4 (5.1) 76.7% female Control group N = 30 Age M = 70.8 (6.6) 76.7% female	Other	The non-face-to-face comprehensive elderly care application, Smart Silver Care, was designed with three key focus areas: physical health, emotional support, and cognitive function improvement.	Waitlist/No-treatment control	Waiting list	8w Individual	–	Depression Korean version of the Short Form Geriatric Depression Scale (SGDS-K) Cognitive Function Korean version of the Mini-Mental State Examination 2^nd^ edition (K-MMSE–2)	Pre, Post (8w)	The results showed no significant changes were found in depression or quality of life between the experimental and control groups. The application was well-received, with high usability and satisfaction scores among participants
Ishihara et al., 2018, Japan	Positive Photo Appreciation N = 28 Age M = 74.3 (5.9) 71.4% female Photo Correspondence Education N = 27 Age M = 73.0 (5.0) 70.4% female	Other	The Positive Photo Appreciation (PPA) Program included 12 weekly 1.5-hour sessions combining photography, appreciation, and collage creation every four sessions. Daily homework focused on recalling enjoyable scenes and practicing gratitude to foster positive emotions.	Non-specific factor component control	Photo Correspondence Education (PCE)	12w Group	–	Depression Centre for Epidemiologic Studies-Depression Scale (CES-D)	Pre, Post (12w)	The study showed a significant reduction in depressive symptoms and increased positive emotions in the Positive Photo Appreciation group. The program was feasible, with high participant satisfaction and no adverse events reported.
Jiang et al., 2024, China	Intervention group: Tele -NT N = 97 Age M = 73.44 (6.59) 70.1% female Intervention group: Tele -EP N = 5 Age M = 77.67 (7.32) 71.6% female TAU N = 95 Age M = 77.42 (6.95) 73.7% female	Psychology-based	Telephone-delivered wisdom enhancement narrative therapy-based intervention(Tele-NT) Telephone-delivered empathy-focused intervention (Tele-EP) Both involved eight 30-minute telephone sessions over 4 weeks, led by trained lay therapists.	Treatment as usual	Received regular telephone calls	4w Individual	–	Depressive Patient Health Questionnaire (PHQ–9)	Pre, Post (4w)	The telephone-delivered wisdom-enhancement narrative therapy effectively reduced loneliness compared to an active control group at the 4-week follow-up assessment. However, there was no significant difference between the telephone-delivered empathy-focused intervention and the active control group at the 4-week follow-up.
Joling et al., 2011, the Netherlands	Bibliotherapy N = 86 Age M = 81.8 (3.8) 69.8% female TAU N = 84 Age M = 81.1 (3.5) 77.4% female	Psychology-Based	The CBT-based bibliotherapy intervention involved providing participants with the “Coping with Depression” self-help manual, three 1-hour home visits from a trained nurse, and two follow-up phone calls over 12 weeks, with participants deciding whether to use the materials.	Treatment as usual	TAU	12w Individual	–	Depression Centre for Epidemiologic Studies-Depression Scale (CES-D)	Pre, Post (12w)	Bibliotherapy as a stand-alone intervention for the elderly did not reduce depressive symptoms more than usual care.
Kam-Pui Lee et al., 2021, China	Mindfulness Program N = 101 Age M = 71.3 (7.0) 82,9% female Waiting List N = 104 Age M = 71.8 (8.2) 84.6% female	Psychology-Based	The modified mindfulness-based stress reduction (mMBSR) program, led by trained professionals, included six weekly home practices with adjustments such as shorter daily sessions, 2-hour classes, no full-day retreat, and sitting postures replacing lying-down postures.	Waitlist/No-treatment control	Waiting List	8w Group	16w	Depression 15-item Geriatric Depression Scale (GDS) Cognitive function Montreal Cognitive Assessment 5-minute (MOCA)	Pre, Post (8w), Follow up (16w; GI only)	The intervention improved mental well-being and depressive symptoms in community dwelling older Chinese adults immediately after intervention. The improvement in depressive symptoms seemed to sustain 8 weeks after intervention.
Kawakami et al., 2018, Japan	Spectator Group N = 29 Age M = 74.3 (4.9) 44.8% female Waiting List N = 29 Age M = 74.2 (5.7) 44.8% female	Other	The spectator group was requested to watch professional baseball games for free for two months. In this period, 21 games were held. Participants were instructed to watch at least two games per month; they can watch whenever they wanted, without limits.	Waitlist/No-treatment control	Waiting List	8w Individual	–	Depression Centre for Epidemiologic Studies-Depression Scale (CES-D)	Pre, Post (8w)	Executive function using the reverse Stroop interference rate showed a non-significant trend of improvement in the spectator group compared with the waiting-list group. The spectator group showed a significant reduction in depressive symptoms compared with the waiting-list group.
Klainin-Yobas et al., 2019, Singapore	Mindfulness Program (MAP) N = 28 Age M = 71.3 (5.6) 71.4% female Health Education Program (HEP) N = 27 Age M = 71.4 (6.0) 77.8% female	Psychology-Based	The Mindfulness Awareness Program (MAP) consisted of weekly 40-minute group sessions for three months, followed by monthly sessions for six months, where instructors guided participants in mindfulness practice techniques.	Active comparator	Health Education Program (HEP)	36w Group	–	Depression 15-item Geriatric Depression Scale (GDS–15) Anxiety 20-item Geriatric Anxiety Inventory (GAI) Cognitive Function Modified version of the Mini-Mental State examination (MMSE)	Pre, Post (36w)	Participants in both intervention arms experienced decreases in depressive and anxiety symptoms over the 36 weeks period. There were no statistically significant changes on cognitive function in both groups over the period.
Lai et al. 2019, China	Life story book N = 124 Age M = 77.98 (7.15) 71% female Social program N = 120 Age M = 76.20 (7.65) 71.7% female	Psychology-Based	Life story intervention integrates a person’s social interests and lifestyle (e.g., hobbies) within a life course perspective (e.g., from childhood to adulthood)	Non-specific factor component control	Social program Discussing daily news	6w Individual	26 W	Depression 15-item Geriatric Depression Scale (GDS)	Pre, Post (6 W), Follow up (13w, 26w)	No significant differences between the two groups.
Lee, K., 2023, Korea	Intervention Group N = 28 Age M = 80.39 (2.57) 39% female Control Group N = 29 Age M = 79.1 (3.9) 52% female	Mind–body-based	The home-based exergame program was conducted at the participants’ homes for 50 min, three times a week, for eight weeks. The exergame program consisted of a 10 min warm-up, 30 min of exercise, and a 10 min cool-down.	Active comparator	Online education on musculoskeletal health once a week but did not exercise.	8w Individual	–	Depression 15-item Geriatric Depression Scale (GDS–15)	Pre, Post (8w)	The 8-week home-based exergame program was found to have a positive effect on the physical function, fall efficacy, depression, and health-related quality of life in older adults.
Legrand and Mille, 2009, France	Intervention Group N = 6 Age M = 66.2 (2.1) 100% female Control Group N = 6 Age M = 67.5 (2.9) 100% Female	Mind–Body-based	Walking at a higher frequency by breaking this weekly dose of exercise into 3–5 workouts with the goal of accumulating 60 min of weekly walking.	Active comparator	Walking for 1 h once a week	4w Group	8w	Depression 30-item Geriatric Depression Scale (GDS–30)	Pre, Post (4w), Follow up (8w)	Frequent exercise was associated with more pronounced antidepressant effects than infrequent exercise, despite other training parameters being similar. One month after the intervention, the reduction in depressive symptoms had been maintained.
Liao et al., 2018, China	Intervention group N = 55 Age M = 71.84 (7.297) 65.5% female Control group N = 52 Age M = 71.75 (8.201) 57.7% female	Mind–Body-based	The combined music and Tai Chi intervention, delivered as group therapy, involved 24 Yang-style Tai Chi movements accompanied by relaxing Chinese folk music. Each 50-minute session included a 5-minute warm-up, 40 minutes of Tai Chi exercises, and a 5-minute cool-down.	Treatment as usual	Individuals assigned to the control group received routine health education monthly delivered by a community nurse.	13w Group	–	Depression Chinese version of the 30-item Geriatric Depression Scale (GDS–30)	Pre, Post (13w)	Combined music and Tai Chi effectively reduced depressive symptoms among community-dwelling older persons. After controlling for socio-demographic data, the true effect of the intervention in relieving depressive symptoms among community-dwelling older persons was confirmed.
Lwi et al., 2023, USA	Mindfulness-Based Stress Reduction N = 23 Age M = 71.7 (7.7) 52% female Control Group N = 26 Age M = 73.9 (7.4) 58% female	Psychology-Based	Mindfulness-Based Stress Reduction: for 8-week class for 2.5 hours, with a day-long retreat in the 6th week.	Active comparator	Brain Health Education Class. The 8-week Brain Health class also met weekly for 2.5 hours, with a day-long retreat in the 5^th^ week.	8w Group	13w	Depression Geriatric Depression Scale short-form (GDS) Anxiety State–Trait Anxiety Inventory (STAI)	Pre, Post (8w), Follow up (13w)	No significant differences between the two groups.
Makizako et al., 2020, Japan	Exercise group N = 30 Age M = 73.1 (5.3) 53% female Control group N = 29 Age M = 73 (5.9) 52% female	Mind–Body-based	The exercise intervention entailed a multicomponent exercise program consisting of 20 weekly 90-min sessions involving aerobic exercise, muscle strength training, postural balance retraining, and dual-task training.	Non-specific factor component control	Educational control: group attended two 90-min education classes during the six-month trial period. The classes included topics (e.g., traffic safety and disaster prevention).	26w Group	52w	Depression 15-item Geriatric Depression Scale (GDS–15)	Pre, Post (26w) Follow up (52w)	Participants who completed the intervention and were assessed immediately afterwards had significantly decreased GDS–15 scores. However, statistical analyses showed that exercise activities did not lead to a reduction in depressive symptoms.
Marchant et al., 2021, England, France and Spain	Caring Mindfulness-based Approach for Seniors N = 73 Age M = 72.1 (7.6) 64% female Health self-management program N = 74 Age M = 73.3 (6.2) 65% female	Psychology-Based	The Caring Mindfulness-Based Approach for Seniors (CMBAS) adapted the mindfulness-based stress reduction format with a preclass interview, 8 weekly 2-hour group sessions, and a half-day meditation practice in the 6th week, tailored to the needs of older adults.	Active comparator	The Health Self-Management Program (HSMP) was a group-based intervention designed to enhance self-efficacy by teaching problem-solving and decision-making skills for active, healthy living.	8w Group	26w	Depression 15-item Geriatric Depression Scale (GDS–15) Anxiety Trait subscale of State Trait Anxiety Inventory (Trait-STAI)	Pre, Post (8w) Follow up (26w)	The 8-week CMBAS intervention showed no superiority over the control group (HSMP) in reducing subclinical anxiety symptoms. Both interventions had similar effects, and depressive symptoms showed minimal improvement due to low baseline levels.
Moret et al., 2022, Italy	Exergame training N = 38 Age M = 70.13 (3.73) 76% female Control Group N = 19 Age = 71.11 (3.72) 68% female	Mind–Body-based	The Dr. Kawashima’s Body and Brain Exercises, an exergame combining cognitive and physical activities. The games targeted cognitive functions like memory, reaction time, processing speed, and executive functions while involving motor planning and execution through full-motion gameplay.	Waitlist/No-treatment control	Continued daily routine	3w Individual	–	Depression Beck Depression Inventory II (BDI-II)	Pre, Post (3w)	The study showed significant improvements in processing speed, inhibition, and depressive symptoms in the intervention group, but post-hoc adjustments reduced these significances.
Prakhinkit et al., 2014, Thailand	Buddhist walking meditation N = 14 Age M = 74 (1.9) 100% female Sedentary Control N = 13 Age M = 81 (1.7) 100% female	Mind–Body-based	The Buddhist walking meditation program combined aerobic walking with Buddhist meditation, involving rhythmic arm swings synchronized with chanting “Budd” and “Dha,” while practicing mindfulness during the exercise.	Waitlist/No-treatment control	Sedentary control group	12w Group	–	Depression Thai Geriatric Depression Scale (TGDS)	Pre, Post (12w)	Buddhist walking meditation was effective in reducing depression, improving functional fitness and vascular reactivity, and appears to confer greater overall improvements than the traditional walking program
Pynnonen et al., 2018, Finland	Intervention Group N = 105 Age M = 77.02 (1.45) 72% female TAU N = 118 Age M = 76.91 (1.43) 78% female	Multicomponent	The intervention allowed participants to choose from three options: (1) an exercise program led by qualified instructors in municipal gyms, (2) a social activity program with group discussions, creative self-expression, and day trips, or (3) personal counseling tailored to individual concerns.	Treatment as usual	The control group received one counseling session which took place prior to randomization. Controls had access to the usual services offered by the municipality and other service providers.	26w Mixed	52w	Depression 15-item Geriatric Depression Scale (GDS–15)	Pre, Post (26w), Follow up (52w)	The intervention did not alleviate depressed mood. Positive changes over time were observed in loneliness, feelings of melancholy, attachment, and guidance but these occurred independently of the intervention.
Scazufca et al., 2022, Brazil	Intervention group N = 360 Age M = N/A 74.4% female TAU N = 355 Age M = N/A 73.8% female	Multicomponent	The intervention combined usual care with psychosocial support, including psychoeducation on depression management and behavioral activation to encourage meaningful activities and positive interactions. Community health workers delivered the intervention through home sessions, supported by a tablet application.	Treatment as usual	Usual care alone	17w Individual	52w	Depression Patient Health Questionnaire (PHQ–9) Anxiety 7-item Generalized Anxiety Disorder Scale (GAD–7)	Pre, Post (35w), Follow up (52w)	At 35 weeks and follow-up, the intervention group demonstrated a higher depression recovery rate (PHQ–9 < 10) compared to the control group. PHQ–9 scores were lower in the intervention group than in the control group at both 35 and 52 weeks. Anxiety symptom levels (GAD–7 scores) were also lower in the intervention group than in the control group at both follow-up assessments.
Scogin et al., 2014, USA	Cognitive-Behavior Therapy N = 70 Age M = 75.67 (6.94) 88.6% female Minimal support condition (MSC) N = 64 Age M = 74.6 (7.91) 76.6% female	Psychology-Based	Cognitive-Behavior Therapy (Home-delivered CBT for depressive symptoms among rural, diverse, and vulnerable older adults).	Non-specific factor component control	Minimal support condition (MSC): receiving brief weekly telephone calls for three months.	20w Group	–	Depression Hopkins Symptom Checklist–20 (HSCL–20)	Pre, Post (20w)	Home-delivered CBT effectively reduces depressive symptom severity in older adults from vulnerable, diverse, and rural backgrounds. While psychological symptoms improved significantly, somatic symptom improvement showed only a trend.
Scott et al., 2024, Australia	Behavioral Activation program N = 66 Age M = 73.58 (6.81) 67% female Positive Psychology active control N = 69 Age M = 74.12 (6.32) 71% female	Psychology-based	The Behavioral Activation program, included six weekly sessions with psychoeducational materials and activities aimed at enhancing well-being through activity monitoring, values identification, and overcoming barriers to meaningful activities.	Active comparator	Positive Psychology active control (PP)	6w Group	12w	Depression Hospital Anxiety and Depression Scale (HADS) Anxiety Hospital Anxiety and Depression Scale (HADS)	Pre, Post (1w), Follow up (12w)	The study found a reduction in depression symptoms in both groups, sustained at the 12-week follow-up. Anxiety symptoms decreased earlier in the Behavioral Activation program group, stabilizing by 1-week follow-up, while the Positive Psychology group showed the lowest anxiety levels at 12 weeks.
Shahidi et al., 2011, Iran	Laugther yoga N = 20 Age M = 65.5 (4.8) 100% female Exercise Therapy N = 20 Age M = 65.7 (4.2) 100% female Control group N = 20 Age M = 68.4 (6.3) 100% female	Mind–Body-based	Laughter Yoga: combining unconditioned laughter and yogic breathing. It involves various activities like clapping, rhythmic movements, and laughter exercises to stimulate diaphragmatic breathing. Exercise Therapy: Ten sessions of aerobic group exercise, including jogging and stretching.	Missing information	The control group is mentioned but not described in detail. They were part of the randomization process.	10 sessions (Nonspecific) Group	–	Depression 30-item Geriatric Depression Scale (GDS–30)	Pre, Post (10 sessions)	Laughter Yoga was at least as effective as the group exercise program in improving depression and life satisfaction in elderly depressed women.
Shih et al., 2021, China	Mindfulness-Based Cognitive Therapy N = 28 Age M = 69.69 (7.48) 89% female Active control group N = 29 Age M = 70.86 (5.80) 86% female	Psychology-Based	Mindfulness-Based Cognitive Therapy (MBCT). Group had eight 2-hour weekly sessions and a 7-hour full-day retreat. Included guided mindfulness exercises, feedback and discussion, homework review, and psychoeducation.	Active comparator	Active control group received a 1-hour physical exercise and a standardized health education session.	8w Group	–	Depression Hamilton Depression Rating Scale (HAMD)	Pre, Post (8w)	Both interventions decrease depressive symptoms. Only Mindfulness-Based Cognitive Therapy improves Autobiographical Memory Specificity (AMS), rumination, and mindfulness.
Solianik et al., 2021, Lithuania	Taichi group N = 15 Age M = N/A 86.67% female Control group N = 15 Age M = N/A 86.67% female	Mind–Body-based	The intervention group practiced Tai Chi with 8-form Yang-style movements for 60 minutes, twice a week, for a total of 10 weeks. Sessions included warm-up, Tai Chi movements, and cool-down.	Waitlist/No-treatment control	The control group did not receive any intervention and was asked to maintain their daily routines during the 10-week period.	10w Group	–	Depression/Anxiety 14-item hospital Anxiety and Depression Scale (HADS)	Pre, Post (10w)	Ten weeks of Tai Chi improved inhibitory control, mental switching, and visuospatial processing, potentially linked to decreased depressive symptoms and increased BDNF. However, no significant correlations were found between changes in psychoemotional state, BDNF levels, and motor learning.
Srisuwan et al., 2020, Thailand	Cognitive Training N = 40 Age M = 66.2 (4.6) 80% female TAU N = 37 Age M = 65.1 (4.0) 76.3% female	Psychology-Based	The Cognitive Training (CT) intervention used the TEAM-V Program, a multidomain training targeting executive function, attention, memory, and visuospatial skills. It included five 120-minute sessions spaced two weeks apart, with each session focusing on different cognitive domains. Participants were encouraged to complete homework during the intervention.	Treatment as usual	The control group received standard clinical care from their usual health-care professionals.	8w Group	52w	Depression Thai version 14-item hospital Anxiety and Depression Scale (HADS) Anxiety Thai version 14-item hospital Anxiety and Depression Scale (HADS) Cognitive function Montreal Cognitive Assessment 5-minute (MOCA)	Pre, Post (26w), Follow up (52w)	The TEAM-V Program significantly reduced anxiety in healthy older adults at the 52 weeks follow-up compared to the control group. While improvements in depression, cognition, and IADL were not statistically significant, there were positive trends in global cognition, memory, attention, and executive function.
Szczepańska-Gieracha et al., 2021, Poland	Virtual therapy N = 13 Age M = 70.18 (4.87) 100% female Control group N = 12 Age M = 71.25 (4.41) 100% female	Psychology-Based	Virtual Therapeutic Garden (VR) group had an additional intervention using virtual reality. As a virtual reality source, the VRTierOne. Eight sessions of VR therapy, each of them 20 min long. VR therapy was conducted twice a week for a period of four weeks.	Active comparator	General fitness training and psychoeducation	4w Group	6w	Depression 30-item Geriatric Depression Scale (GDS–30) Anxiety Hospital Anxiety and Depression Scale (HADS)	Pre, Post (4w), Follow up (6w)	Immersive virtual therapy with guided imagery in a therapeutic garden significantly reduced depression, anxiety, and stress in older women. While the control group showed no significant changes.
Tabei et al., 2024, Irán	Laughter Yoga N = 31 Age M = 66.06 (4.5) 64.5% female Music Intervention N = 25 Age M = 64.80 (4.2) 64% female TAU N = 28 Age M = 65.86 (3.7) 60.7% female	Mind–Body-based	Laughter Yoga intervention: included two 30-minute weekly sessions combining warm-up exercises, deep breathing, and laughter exercises. Music intervention: involved two 30-minute sessions of calming music at home, chosen in consultation with a professional to align with the participants’ cultural preferences.	Treatment as usual	TAU	8w Group	4w	Depression 30-item Geriatric Depression scale (GDS–30) Anxiety Depression anxiety and stress scale questionnaire (DASS–21)	Pre Post (8w), Follow up (4 w)	Both laughter yoga and music interventions proved effective in improving depression, anxiety, and stress in aged individuals. However, laughter yoga intervention demonstrated a superior effect and better acceptance among elders.
Tanaka et al., 2019, Japan	Cognitive Behavioral Therapy for insomnia N = 23 Age M = 68.8 (8.6) 68.2% female Control group N = 23 Age M = 70.8 (7.6) 80.0% female	Psychology-Based	The CBT-I intervention included sleep hygiene education, stimulus control, sleep restriction, cognitive therapy, and relaxation techniques. It comprised a 60-minute group session, a 45-minute individual session, and two follow-up phone sessions (20 minutes each) at 1 week and 1 month.	Waitlist/No-treatment control	Received no contact during the follow-up period but were free to seek general information about sleep hygiene.	3w Group	–	Depression Japanese version of the Geriatric Depression Scale short form (GDS-SF).	Pre, Post (13w)	A Cognitive Behavioral Therapy for insomnia intervention effectively reduced depressive symptoms in older adults, showing a significant decrease in GDS-SF scores compared to the control group. The effect size for symptom reduction was small (Cohen’s d = 0.34).
Titov et al., 2015, Australia	Internet-delivered Cognitive Behavioral Therapy N = 29 Age M = 64.52 (2.58) 81.5% female Control group N = 25 Age M = 66.16 (3.80) 64.0% female	Psychology-Based	The Internet-delivered Cognitive Behavioral Therapy (iCBT) intervention, Is an 8-week program featuring five online lessons, summaries, homework, automated reminders, secure therapist messaging, and case stories of older adults recovering from depression, based on evidence-based CBT principles.	Waitlist/No-treatment control	Delayed-treatment waitlist control group.	8w Individual	52w	Depression 9-item Patient Health Questionnaire (PHQ–9) Anxiety Generalized Anxiety Disorder 7-item Scale (GAD–7)	Pre, Post (8w) Follow up (13w, 52w)	The treatment group showed significant reductions in depression and anxiety symptoms compared to the waitlist control group, with sustained improvements at 13 weeks and 52 weeks follow-ups.
Titov et al., 2016, Australia	Interview plus clinician-guided group (ICG) N = 153 Age M = 65 (3.8) 65% female Interview plus self-guided group (ISG) N = 140 Age M = 66 (4.6) 64% female Self-guided group (SG) N = 140 Age M = 67(5.6) 63% Female	Psychology-Based	Interview plus clinician-guided group (ICG): The Wellbeing Plus Course is an 8-week internet-delivered intervention for older adults, focusing on cognitive challenging, exposure, and behavioral activation to reduce depression and anxiety. It includes five online lessons, homework, and clinician support through brief scripted phone calls and weekly contact.	Active comparator	Participants in the Interview plus self-guided group (ISG) and Self-guided group (SG) groups were informed via the online application system that they would not receive clinical contact throughout treatment unless their symptoms became more severe.	8w Individual	13w	Depression 9-item Patient Health Questionnaire (PHQ–9) Anxiety Generalized Anxiety Disorder 7-item Scale (GAD–7)	Pre, Post (8w), Follow up (13w)	The study found significant reductions in depression and anxiety symptoms across all groups, with no differences between them. The transdiagnostic Wellbeing Plus Course, delivered with or without clinician guidance, was effective and well-accepted by older adults, showing high completion rates, satisfaction, and significant clinical benefits.
Tran et al., 2023, Canadá	Intervention group N = 14 Age M = 71.43 (9.0) 57.1% female Waitlist N = 13 Age M = 75.31 (9.5) 92.3% female	Psychology-based	Mindfulness-Based Stress Reduction (MBSR). This program was established in 1979 by Kabat-Zinn. Consisted of nine sessions, including orientation, were three-hours in duration; a tenth session consisted of an all-day retreat of mindfulness practice.	Waitlist/No-treatment control	Waitlist	8w Group	13w	Depression Patient Health Questionnaire (PHQ–9) Anxiety Geriatric Anxiety Inventory (GAI)	Pre, Post (8w), Follow up (13w)	Mindfullness-Based Stress Reduction improved anxiety scores for patients with early cognitive deficits postintervention.
Van der Weele et al., 2012, Dutch	Coping with Depression course N = 121 Age M = 80.96 (4.552) 70% female TAU N = 118 Age M = 81.24 (5.239) 75% female	Psychology-Based	The Coping with Depression course, based on CBT, included 10 weekly group sessions with 2 instructors and 6–10 participants. For those unable to attend group sessions, the course was offered individually at home.	Treatment as usual	To ensure usual care.	10w Group	52w	Depression Montgomery-Åsberg Depression Rating Scale (MADRS)	Pre, Post (26w), Follow up (52w)	The stepped-care intervention programme for screen positive depressed subjects did not result in better clinical outcomes compared with usual care. Economic evaluation of the stepped-care intervention programme also showed no favourable results.
Wahbeh et al., 2016, USA	Internet Mindfulness Meditation Intervention N = 8 Age M = 76.9 (9.0) N/A% female Internet Education N = 8 Age M = 75.5 (5.6) N/A% female	Psychology-Based	The Internet Mindfulness Meditation Intervention (IMMI) included weekly one-hour online sessions on stress management, mindfulness practices (e.g., body scan, sitting meditation), and daily activities, along with 30 minutes of daily home practice. The goal was to help older adults manage stress and improve self-care.	Active comparator	Internet Education was matched for session time and home practice. The internet sessions included a general health video and questions about the material. For home practice, the participants listened to podcasts about the same topic.	6w Individual	–	Depression Centre for Epidemiologic Studies Depression Scale (CESD–5)	Pre, Post (6w)	The results showed no significant differences in mood, stress, sleep quality, or cognitive outcomes were observed between the two groups, as expected due to the small sample size. Both interventions demonstrated high acceptability and adherence.
W. L. Wang et al., 2023, China	Magic intervention N = 19 Age M = 72.1 (7.6) 73.7% female Control group N = 19 Age M = 71.8 (6.5) 68.4% female	Other	The Magic intervention program, tailored for older adults, included 90-minute sessions twice weekly for 6 weeks, starting one week after initial mental health education. It integrated magic-based activities to develop psychosocial skills, similar to skill training and social activity interventions.	Waitlist/No-treatment control	Maintained their regular routines and daily activities.	6w Group		Depression 15-item Geriatric Depression Scale (GDS–15)	Pre, Post (6w)	The magic intervention significantly increased self-esteem and reduced depressive symptoms with large effect sizes. A moderate negative correlation was observed between post-intervention self-esteem and depressive symptoms in the magic intervention group.
C. X. Wang et al., 2023, China	Solution-focused group counseling N = 107 Age M = 71.59 (7.70) 64% female Control group N = 85 Age M = 73.39 (6.88) 64% female	Psychology-based	The Solution-Focused Group Counseling (SFGC) intervention, based on Solution-Focused Brief Therapy (SFBT), included bi-weekly 90-minute sessions over 6 months. It comprised four stages: building relationships (sessions 1–3), work (4–7), change (8–10), and finalization (11–12).	Waitlist/No-treatment control	Waitlist-No treatment	24w Group	–	Depression 10-item version of the Center for Epidemiological studies Depression Scale (CES-D)	Pre, Post (24w)	Significant group-by-session effect indicated different changing patterns of depressive symptoms from pretest to posttest in the two groups. T-tests and effect sizes showed that depressive symptoms of participants in the intervention group decreased slightly, but not in the control group.
Xie et al., 2019, China	Behavioral activation N = 40 Age M = 71.95 (3.922) 57.5% female TAU N = 40 Age M = 71.85 (3.725) 60.0% female	Psychology-based	The Behavioral Activation (BA) intervention followed three main steps: activity monitoring, scheduling, and modification. Over 8 sessions, assignments were reviewed and repeated multiple times to accommodate the low education levels of left-behind elders.	Treatment as usual	The regular care included regular physical examinations, and reviewing the current health symptoms same education contents delivered by village doctors.	8w Group	13w	Depression 30-item Geriatric Depression Scale (GDS–30) Anxiety Beck Anxiety Inventory (BAI)	Pre, Post (8w), Follow up (13w)	After 8 sessions of Behavioral Activation, the intervention group showed significant reductions in GDS and BAI scores compared to the control group. These improvements were sustained at the 13 weeks follow-up, while the control group showed no significant changes in BAI scores.
Yujie Ge et al., 2021, China	Tai-Chi Group N = 32 Age M = 70.2 (5.4) 34.3% female TAU N = 33 Age M = 72.9 (6.6) 51.5% female	Mind–Body-Based	Tai-Chi Group (TCG). Subjects received 3 sessions/ week, 60 min per session, with a duration of 8 weeks.	Treatment as usual	TAU	8w Group	–	Depression 30-item Geriatric Depression Scale (GDS–30)	Pre, Post (8w)	The Tai-Chi exercise could improve physical function (the lower limbs’ strength and gait speed), fear of falling, and depression.

### Depression and anxiety instruments

Most studies measured changes in depressive symptoms assessed by the GDS (=35), with seven using PHQ-9, six using CES-D, five using HADS, three using BDI, and one using DASS (the depression subscale), MADRS, and HSCL-90, respectively.

Similarly, for anxiety changes, 14 studies used GAD-7, three studies used STAI (only the state subscale was extracted), four used DASS, five used HADS, one used the SCL-90 (the anxiety subscale), and three studies used BAI (See Table 1).

### Intervention effectiveness on depressive symptom reduction

Interventions targeting depressive symptoms showed a significant moderate effect compared to controls (*k* = 60; *d* = −0.530; *p* < .001) pre-to-post. When removing two outliers, the effect remained significant although it was somewhat lower (*k =* 58*; d =* −0.474; *p* < .001*).*

There was also a significant small-to-moderate effect comparing symptom levels pre-to-follow-up (*k* = 24; *d* = −0.386; *p* = .002; Supplementary file 3). Given the large heterogeneity (*I* ^2^ = 84.51 for pre-to-post and *I* ^2^ = 87.24 for pre-to-follow-up), several factors were examined as potential moderators of these findings. Specifically, for pre-to-post and pre-to-follow-up depressive levels, the findings revealed an effect of mean age, with younger participants showing higher reductions of depressive symptoms when receiving an intervention (*b* = 0.055; *p* < .001, *b* = 0.075; *p* < .007). Targeting depression as the primary outcome also seems to moderate both pre-to-post and pre-to-follow-up comparisons (*b* = −0.313; *p* < .042, *b* = −0.490; *p* < .036). On the other hand, when comparing intervention types (psychology-based, mind–body-based, multicomponent, other), no significant differences emerged. Likewise, there was no difference when comparing types of control (specific, TAU, waitlist, other nonspecific; Table 2) across both pre-to-post and pre-to-follow-up comparisons.

**Table 2.** Single precision-weighted regression models

	Pre versus post comparisons				Pre versus follow-up comparisons			
	*k*	*b*	*SE*	*p*	*k*	*b*	*SE*	*p*
Depression								
Cognitive function change	13	0.411^f^	0.073	<.001	7	0.571	0.392	.145
Control type^a^	59				24			
Specific		0.312	0.273	.253		−0.392	0.501	.434
Treatment as usual		0.229	0.241	.344		−0.576	0.456	.207
Waitlist		−0.092	0.258	.722		−0.524	0.630	.406
Follow-up time	–	–	–	–	24	0.004	0.003	.288
Intervention type^b^	60				24			
Psychology-based		0.040	0.257	.876		0.048	0.547	.930
Mind–body-based		−0.454	0.297	.126		−0.845	0.656	.198
Other		−0.135	0.334	.685		−0.714	0.799	.372
Mean age	56	0.055	0.017	.001	22	0.075	0.029	.007
Percentage of women within sample	59	−0.007	0.005	.112	24	−0.010	0.008	.176
Primary outcome^c^	60	−0.313	0.154	.042	24	−0.490	0.234	.036
Publication year	60	0.019	0.019	.318	24	0.001	0.039	.991
Quality^d^	60				24			
Moderate		−0.224	0.169	.185		−0.427	0.411	.298
Strong		−0.167	0.196	.393		−0.353	0.272	.194
Treatment setting^e^	60				24			
Individual		0.068	0.163	.679		0.057	0.322	.859
Mixed		0.551	0.537	.305		–	–	–
Anxiety								
Cognitive function change	10	0.045	0.195	.817	7	0.263	0.314	.401
Control type^a^	30				19			
Specific		0.041	0.321	.897		0.116	0.462	.802
Treatment as usual		−0.259	0.298	.384		−0.210	0.445	.637
Waitlist		−0.537	0.351	.126		–	–	–
Follow-up time	–	–	–	–	19	<0.001	0.002	.950
Intervention type^b^	30				19			
Psychology-based		−0.427	0.247	.084		−0.138	0.200	.491
Mind–body-based		−0.607	0.363	.094		−0.639	0.397	.108
Other		−0.233	0.380	.540		−0.489	0.405	.227
Mean age	27	0.038	0.016	.020	17	0.033	0.021	.126
Percentage of women within sample	30	0.003	0.005	.553	19	0.002	0.005	.759
Primary outcome^c^	29	−0.093	0.169	.579	19	−0.028	0.161	.864
Publication year	30	0.017	0.030	.582	19	−0.018	0.035	.607
Quality^d^	30				19			
Moderate		0.190	0.199	.340		0.237	0.226	.295
Strong		0.140	0.198	.480		−0.052	0.164	.750
Treatment setting^e^	30				19			
Individual		−0.147	0.175	.400		−0.051	0.166	.760
Mixed		0.455	0.385	.238		0.444	0.255	.082

a Referenced to nonspecific controls.

b Referenced to multicomponent therapy.

c 0 = No, 1 = Yes.

d Referenced to weak quality.

e Referenced to group treatment.

f sensitivity analyses show that this effect is driven by a leverage point – removal of one outlier, yielding a 7.5 more extreme value compared to its adjacent value leads to nonsignificance of this effect (*k* = 12; *b* = 0.135; *SE* = 0.156: *p* = .386).

A potential effect of cognitive function change was reported for pre-to-post comparisons but lost significance when two outliers were removed. There was no significant effect of any other potential moderator for the depressive symptoms change from pre-to-post or from pre-to-intervention to follow-up.

### Intervention effectiveness on anxiety symptom reduction

For anxiety levels, the findings showed a small-to-moderate effect of interventions regarding pre-to-post changes compared to controls (*k* = 30; *d* = −0.333; *p* < .001) and a small, albeit nominally nonsignificant effect for pre-to-follow-up changes (*k* = 19; *d* = −0.205; *p* = .052). When examining potential moderators due to heterogeneity (*I* ^2^ = 81.92 for pre-to-post and *I* ^2^ = 79.38 for pre-to- follow-up), similarly to depression, younger participants showed higher symptom reduction for pre-to-post (*b* = 0.038; *p* = 0.020). No significant effects were observed for type of intervention, type of controls, or any other of the examined variables, both for pre-to-post and pre-to-follow-up changes in anxiety symptoms (Table 2).

### Dissemination bias

Begg and Mazumdar’s rank correlation method indicated some evidence for funnel plot asymmetry in both anxiety calculations and in pre-to-post comparisons for depression. Moreover, trim-and-fill suggested asymmetry in the anxiety follow-up and Sterne and Egger’s regression in both depression calculations (see Supplementary file 5). All other methods were inconspicuous for the four meta-analytical subsets. In all, these results indicate that the calculated summary effects may be somewhat affected by effect inflation, but the possible confounding effects appear not to be substantial.

### Study quality

Based on the EPHPP ratings, there were 11 studies with strong quality, 24 with moderate, and 23 with weak quality (Supplementary file 4). Dummy-coded precision-weighted single meta-regressions with low-quality studies as reference compared to moderate- and strong-quality studies did not show any systematic influences of study quality on the effect sizes for depression or anxiety. However, specification curves showed somewhat stronger summary effects for qualitatively weak studies in anxiety. Based on the GRADE system, certainty of evidence for both depressive and anxiety symptoms was moderate for psychology-based, mind–body-based, and other interventions, whereas it was low for multicomponent interventions. Certainty of evidence was downgraded mostly due to high unexplained heterogeneity. Further details can be found in Supplementary File 4.

### Multiverse analyses findings

#### Specification curve

**Figure 2.**
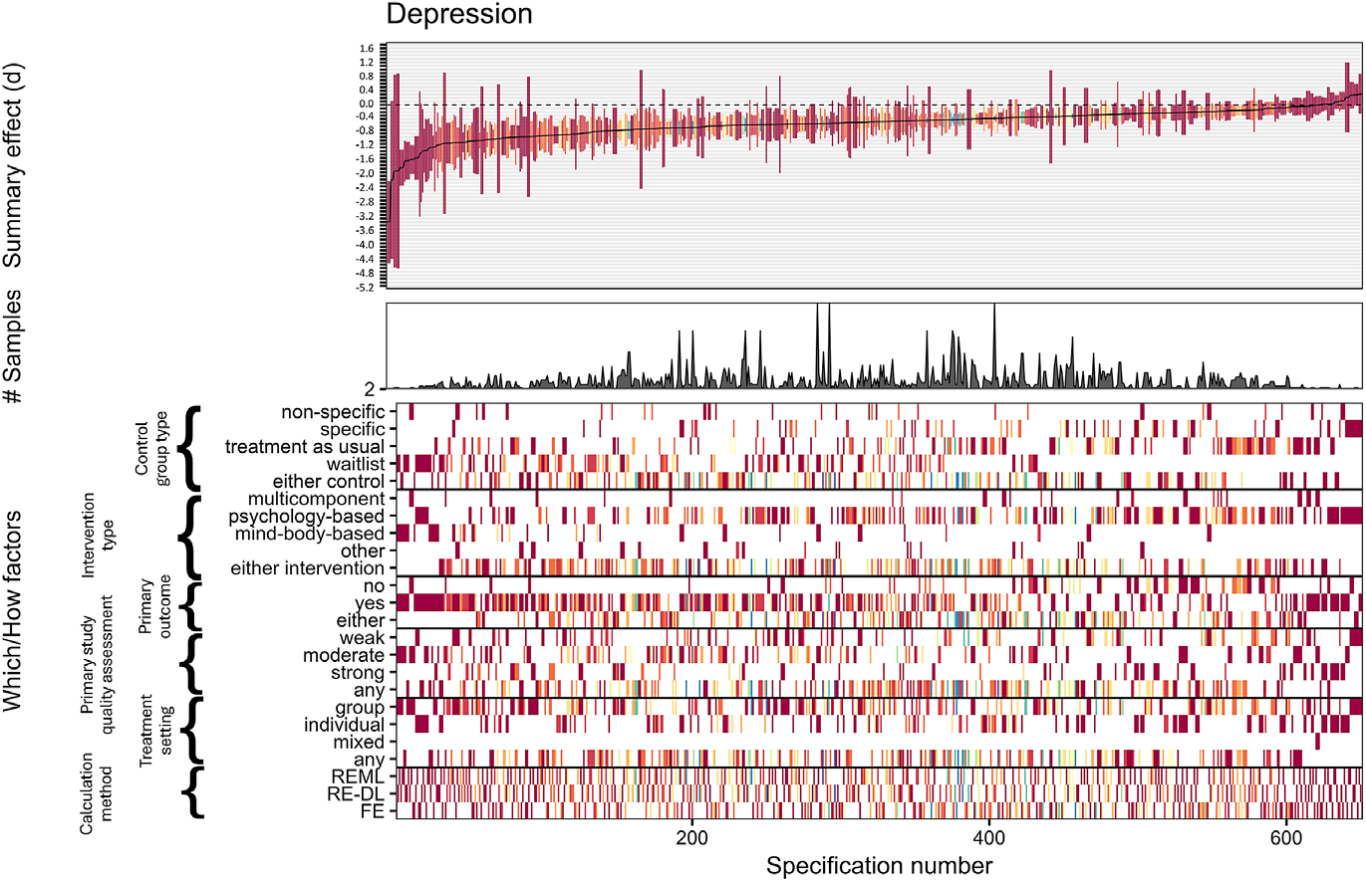
Effect sizes for depressive symptoms specification curve.

Out of 651 reasonable specifications for depression, 444 (68%) yielded significant negative summary effects, thus indicating treatment effectiveness. Merely 29 summary effects were positive, none of which reached nominal statistical significance. Treatment effects averaged *d* = −0.529 (*Md* = −0.475) with 50% of the most typical summary effect estimates (i.e., interquartile range) ranging from −0.701 to −0.264, thus suggesting a remarkable generality of the observed treatment effect. Effect sizes of more accurate estimates (i.e., with larger precision and smaller confidence intervals) appear to indicate numerically larger effects of mind–body-based interventions as well as waitlist controls (Figure 2). Removing two outliers from these analyses yielded somewhat numerically lower summary effect averages, yielding *d* = −0.463 (*Md* = −0.455, interquartile range = −0.634 to −0.242), but the effect generality and robustness of the observed meaningful treatment effect remained unaffected (Supplementary file 6). Depression follow-up results were similar in terms of interpretation to the pre-to-post comparisons, although effect sizes were numerically weaker. Out of 210 reasonable specifications, 80 (38%) yielded significant negative summary effects, while only 14 specifications led to positive effects, none of which were significant. Effects averaged at *d* = −0.356 (*Md* = −0.310) with an interquartile range from −0.476 to −0.119 (Supplementary file 7).

**Figure 3.**
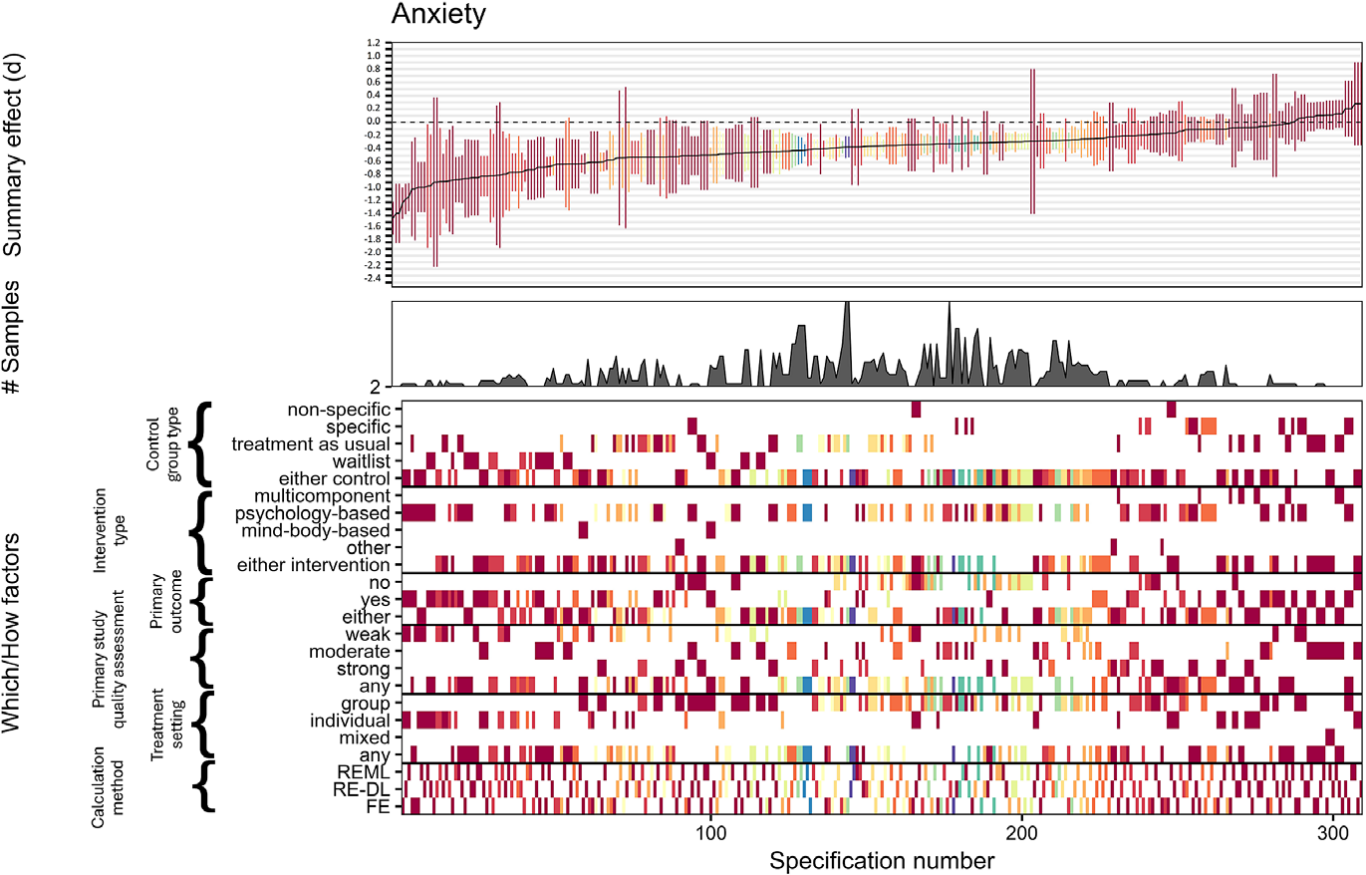
Effect sizes for anxiety specification curve.

For anxiety, out of 309 reasonable specifications, 190 (61%) yielded significant negative summary effects with only 22 specifications leading to positive effects, all of which failed to reach significance. Summary effects averaged at *d* = −0.391 (*Md* = −0.354) with an interquartile range from −0.525 to −0.210. There was some indication that primary studies that had been rated to have weaker quality tended to show somewhat stronger effects, thus tentatively conforming to the results from our publication bias analyses. However, despite this observation, summary effect calculations showed in this case once more a remarkable generality of the treatment effect (Figure 3). Anxiety follow-up calculations supported results from pre-to-post comparisons, although, as expected, the effects were once again weaker. Out of 123 reasonable specifications, 78 (63%) yielded significant negative effects with 10 specifications leading to positive albeit nonsignificant summary effects. Summary effects averaged *d* = −0.265 (*Md* = −0.272) and the interquartile range showed values from −0.343 to −0.198 (Supplementary file 8).

#### Combinatorial meta-analysis

Results from combinatorial meta-analyses were broadly in line with findings from our specification curve calculations, supporting the generality of our observed treatment effects. Depression analyses indicated an average effect size of *d* = −0.532 (*Md* = −0.532) and an interquartile range from −0.587 to −0.477 (Supplementary file 9). Removal of two outliers led to numerical changes with *d* = −0.475 (*Md* = −0.475; interquartile range = −0.523 to −0.426; Supplementary file 10). Depression follow-up calculations showed again numerically smaller but nonetheless meaningful effects (*d* = −0.421; *Md* = −0.420; interquartile range = −0.511 to −0.328; Supplementary file 11).

Anxiety analyses indicated effect averages of *d* = −0.359 (*Md* = −0.261) and an interquartile range from −0.423 to −0.292 (Supplementary file 12). Follow-up analyses showed an average of *d* = −0.386 (*Md* = −0.390; interquartile range = −0.480 to −0.279; Supplementary file 13). GOSH-plots did not indicate systematic patterns for depression or anxiety.

## Discussion

This study provides the most comprehensive synthesis to date of randomized controlled trials (RCTs) evaluating preventive psychosocial interventions that aim to reduce subclinical depressive and anxiety symptoms in older adults. The meta-analysis includes primary studies that examine different intervention and control types and assesses both depression and anxiety outcomes to estimate prevention effectiveness against the most prevalent CMDs. A broad number of studies from different geographical regions is included, thus encompassing numerous samples experiencing their senior years in a variety of sociocultural contexts. Publication bias and multiverse analyses allowed for assessment of potential effect inflation and effect generality across potentially moderating variables and their interactions, allowing for robust conclusions.

Specifically, the findings indicate that such interventions are effective for reducing the participants’ depressive symptoms immediately after the intervention and at follow-up, thus demonstrating that the prevention effect is sustained in the long term. Findings are similar for anxiety; however, the prevention effects seem to be less sustainable at follow-up. Importantly, the observed effectiveness is independent of intervention or control condition types, suggesting that any preventive intervention is indeed effective for protecting older adults against CMDs. Only age seems to moderate these findings, with younger participants benefiting more substantially from preventive interventions. Moreover, mind–body-based interventions appeared to be somewhat superior and more effective when comparing interventions with waitlist controls. These results are robust and largely unaffected from publication bias and study quality, although the high unexplained heterogeneity observed somewhat limits the certainty of recommendations across the various intervention types.

This is the first meta-analysis to examine the generality of the intervention effect and the only one that accounts for different moderator effects and their interactions so far. Our findings are broadly in line with results of the only prior meta-analysis on preventive interventions against depression for older adults that encompassed all types of psychosocial interventions to date (Forsman et al., 2011). We update, expand, and refine these findings further; specifically, we included only trials that were randomized; we doubled the number of included studies with a wider sample; we included anxiety as an outcome to better encompass the CMDs in older adults; and we performed advanced meta-analytic procedures to refine the effectiveness effect size and to account for several moderators. This may also explain why our findings contrast with their results regarding social activity-based intervention effects, as we did not obtain superior results to other interventions. In fact, intervention effects showed remarkable generality across intervention types, with evidence suggesting somewhat more beneficial effects of mind–body-based interventions. This is consistent with recent studies that have supported the relevance of physical exercise to reducing depressive symptoms (Kim, Hong, & Choi, 2022) and to improving health and well-being outcomes for older adults (Kappen, Pejman, & Nacke, 2019). However, determining which specific elements of mind–body-based interventions are responsible for these superior effects is challenging, because such interventions include a large variety of physical activities that differ in terms of intensity, modality (that is group-individual), frequency, or purpose. Furthermore, it seems that the effect sizes of more accurate estimates (that is smaller CIs) derive from studies that compare the intervention groups with waitlist controls, in line with previous findings indicating that waitlist controls groups are less effective than other types of control conditions, such as TAU (Plessen, Karyotaki, Miguel, Ciharova, & Cuijpers, 2023).

Compared to depression, evidence for preventive interventions against anxiety is more limited and inconsistent, although there has been growth of the relevant literature in the last decade. Our results align with those of a recent meta-analysis examining the effect of remote CBT on self-reported anxiety (Ando et al., 2024) and contrast those of Dworschak et al. (2022), where internet-based interventions did not seem to reduce anxiety symptoms. However, both meta-analyses were based on a limited number of primary studies ranging from two to six. Thus, the present meta-analysis based on 25 studies (that is, an amount four to twelve times larger) is the first to offer a more precise estimation of the effect on anxiety, encompassing a variety of intervention types and modalities.

Notably, age was the only factor moderating the intervention effectiveness on depressive and anxiety symptoms, as younger participants seem to benefit more from such interventions. A potential explanation for this finding may be related to how the decline in memory and learning capacity that come with age hinders the skills acquisition aimed at through the interventions. However, the exact impact of such decline on intervention outcomes is currently unknown (Wuthrich et al., 2024). Also, our analyses indicated no meaningful impact of mild cognitive decline on the interventions’ effectiveness, suggesting that other aging-related effects may be responsible for decreasing intervention effectiveness.

Despite the strengths of our study, the findings should be interpreted having in mind the following limitations. First, despite considering a comprehensive number of databases, we only included studies reported in English and Spanish, potentially introducing a language bias. However, the presently used publication bias detection methods should have picked up on publication language-based biases and indicate that such influences are modest at best. Second, given the focus on prevention studies, there is a possibility of a floor effect due to low symptom levels; this may suggest that in fact the intervention effectiveness may be even larger than detected. Third, there were only few studies including results based on intention to treat samples; thus, a completer-only bias may have influenced the findings. Fourth, the observed age moderation effect may be due to sample composition rather than genuine age differences in responsiveness to interventions, as there may have been an overrepresentation of younger participants, a common phenomenon observed in intervention studies focusing on the elderly. Fifth, it was not possible to define the effect size of specific intervention types (that is, CBT, yoga, mindfulness, etc.) due to the limited number of studies focusing on specific interventions and the heterogeneity among them in terms of procedures and format.

Given the overall moderate confidence on the observed effects of the included intervention types, continued investment in high-quality, large-scale RCTs is essential to guide policy and implementation. Future research should focus on the detection of the specific mechanisms that underlie psychosocial interventions, to determine how to optimize their impact, as this is a common gap in the literature addressing interventions destined to older adults (Iwano, Kambara, & Aoki, 2022; Qiu et al., 2025). This may be particularly relevant in the context of digital and low-support interventions that seem to emerge as a potential future modus operandi (Wuthrich et al., 2024) in service provision. Importantly, given the exclusion of studies focusing on populations with physical illnesses, the high prevalence of mental symptoms in such populations (Berk et al., 2023), and the comorbidity of such conditions in the elderly (Devita et al., 2022), future endeavors should address this gap to guide the implementation of preventive strategies for older people with physical conditions and CMD symptoms.

According to our findings, preventive psychosocial interventions seem to be particularly beneficial for older individuals applied in a variety of delivery formats and settings, independent of its content or other characteristics. Given that the interventions seem to be more effective with younger participants, there is a need for primary care and mental health care professionals to reinforce early screening for depressive and anxiety symptomatology, to prevent further worsening of these conditions.

In conclusion, the present results clearly indicate the importance of early, nonpharmacological approaches in mental health prevention for aging populations. Low-cost and low-complexity preventive interventions would be a strong candidate for providing a cost-effective solution for common mental illness symptoms. Importantly, such interventions could be included in the main agenda of primary health care services and become part of routine care provision for older adults, if the aim is to protect them against the impact of mental disorders and to promote positive aging.

## Supporting information

10.1017/S0033291726104607.sm001 Saldivia et al. supplementary material Saldivia et al. supplementary material

## Data Availability

Data and all other relevant materials are publicly available either at the website of Open Science Framework (www.osf.io) or upon request to the corresponding author.

## Supplementary material

The supplementary material for this article can be found at http://doi.org/10.1017/S0033291726104607.
