# Supplementary material for: A meta-analysis of preventive psychosocial interventions against depressive and anxiety symptoms in older adults
Source: Psychol Med. 2026 May 14;56:e151. doi: 10.1017/S0033291726104607 (PMC13200161; doi:10.1017/S0033291726104607)
Supplement: Saldivia et al. supplementary material [file S0033291726104607sup001.zip › Supplementary File 2 _search_strategy.docx]

**Supplementary File 2**

Search strategies

TITLE-ABS-KEY

("Psychosis" OR “Psychoses” OR "Psychotic" OR “severe mental illness” OR “schizophrenia”)

AND TITLE-ABS-KEY (“Quality of Life” OR “QoL”)

AND

TITLE-ABS-KEY (“intervention” OR “therapy” OR “training”)

AND

TITLE-ABS-KEY (“Randomised” OR “Randomized” OR “RCT”)

The specific search string was:

(elder* OR ag*ing OR "old*people" OR "old*person*" OR "old* adult*" OR senior* OR pensioner* OR retire* OR "old age" OR "late* life" OR "geriat*")

AND (anxi* OR depress* OR stress* OR distress* OR affective OR mood OR "mental health" OR emotion*)

AND ("intervention" OR "therapy" OR "training "OR "program*")

AND ("Randomized" OR "Randomized" OR "RCT")

NOT ("review" OR "meta-analysis" OR "meta-analysis")

NOT ("drug" OR "pharmacolog*" OR "serotonin" OR "electroconvulsive" OR "antidepress*" OR "anti-anxi*" OR "medication*" OR "medical patient*" OR "medical therapy" OR "medical treatment*").
