# Supplementary material for: A meta-analysis of preventive psychosocial interventions against depressive and anxiety symptoms in older adults
Source: Psychol Med. 2026 May 14;56:e151. doi: 10.1017/S0033291726104607 (PMC13200161; doi:10.1017/S0033291726104607)
Supplement: Saldivia et al. supplementary material [file S0033291726104607sup001.zip › Supplementary File 3 Summary effects.docx]

Supplementary File 3. Summary effects for pre-post changes in treatment-controlled designs in depression, anxiety, and cognitive function.

|  | *k* | *n* | *I*² | Cohen *d* | *p* | *LCI* | *UCI* |
| --- | --- | --- | --- | --- | --- | --- | --- |
| Pre vs. post comparisons | | | | | | | |
| Depression | 60 | 5,517 | 84.51 | -0.530 | <.001 | -0.675 | -0.384 |
| Anxiety | 30 | 2,999 | 75.41 | -0.369 | <.001 | -0.527 | -0.211 |
| Cognitive Function | 13 | 1,075 | 94.13 | 0.185 | .496 | -0.348 | 0.719 |
| Pre vs. follow-up comparisons | | | | | | | |
| Depression | 24 | 2,449 | 87.24 | -0.386 | .002 | -0.632 | -0.143 |
| Anxiety | 19 | 2,080 | 58.59 | -0.241 | .001 | -0.389 | -0.093 |
| Cognitive Function | 7 | 428 | 61.34 | 0.574 | .001 | 0.234 | 0.913 |
