# Supplementary material for: A meta-analysis of preventive psychosocial interventions against depressive and anxiety symptoms in older adults
Source: Psychol Med. 2026 May 14;56:e151. doi: 10.1017/S0033291726104607 (PMC13200161; doi:10.1017/S0033291726104607)
Supplement: Saldivia et al. supplementary material [file S0033291726104607sup001.zip › Supplementary File 4 Quality assesment.docx]

**Supplementary File 4**

**Quality assessment of the included RCTs based on the EPHPP criteria**

| Authors | Primary Intervention type | Selection bias | Study design | Confounders | Blinding | Data collection methods | Withdrawals/  dropouts | Global rating |
| --- | --- | --- | --- | --- | --- | --- | --- | --- |
| Joling et al.,2011 | Psychology-based | Moderate | Strong | Strong | Moderate | Strong | Moderate | Strong |
| Marchant et al.,2021 | Psychology-based | Moderate | Strong | Strong | Moderate | Strong | Strong | Strong |
| Xie et al.,2019 | Psychology-based | Moderate | Strong | Strong | Moderate | Strong | Strong | Strong |
| Lai et al.,2019 | Psychology-based | Moderate | Strong | Strong | Moderate | Strong | Moderate | Strong |
| Titov et al.,2016 | Psychology-based | Moderate | Strong | Strong | Moderate | Strong | Strong | Strong |
| Srisuwan et al.,2020 | Psychology-based | Moderate | Strong | Strong | Moderate | Strong | Strong | Strong |
| Denkova et al., 2024 | Psychology-based | Weak | Strong | Strong | Moderate | Strong | Strong | Moderate |
| Cieślik et al.,2023 | Psychology-based | Weak | Strong | Strong | Moderate | Strong | Strong | Moderate |
| Casemiro et al.,2018 | Psychology-based | Moderate | Strong | Weak | Moderate | Strong | Strong | Moderate |
| Chan and Lo,2023 | Psychology-based | Weak | Strong | Strong | Moderate | Strong | Strong | Moderate |
| Kam‐Pui Lee et al.,2021 | Psychology-based | Moderate | Strong | Strong | Weak | Strong | Strong | Moderate |
| Klainin-Yobas et al.,2019 | Psychology-based | Weak | Strong | Strong | Moderate | Strong | Moderate | Moderate |
| Jiang et al., 2024 | Psychology-based | Weak | Strong | Strong | Moderate | Strong | Strong | Moderate |
| Tanaka et al.,2019 | Psychology-based | Weak | Strong | Strong | Moderate | Strong | Strong | Moderate |
| Titov et al.,2015 | Psychology-based | Moderate | Strong | Strong | Weak | Strong | Strong | Moderate |
| Shih et al.,2021 | Psychology-based | Weak | Strong | Strong | Moderate | Strong | Strong | Moderate |
| Szczepańska-Gieracha et al.,2021 | Psychology-based | Weak | Strong | Strong | Moderate | Strong | Strong | Moderate |
| Wahbeh et al.,2016 | Psychology-based | Weak | Strong | Strong | Moderate | Strong | Strong | Moderate |
| Tran et al., 2023 | Psychology-based | Weak | Strong | Strong | Moderate | Strong | Strong | Moderate |
| Lwi et al.,2023 | Psychology-based | Weak | Strong | Weak | Weak | Strong | Moderate | Weak |
| Almeida et al.,2020 | Psychology-based | Weak | Strong | Strong | Weak | Strong | Moderate | Weak |
| Ayudhaya et al.,2020 | Psychology-based | Weak | Strong | Weak | Moderate | Strong | Strong | Weak |
| Calatyud et al.,2021 | Psychology-based | Moderate | Strong | Weak | Moderate | Strong | Weak | Weak |
| Chan et al.,2013 | Psychology-based | Weak | Strong | Weak | Weak | Strong | Strong | Weak |
| Dear et al.,2014 | Psychology-based | Weak | Strong | Weak | Weak | Strong | Strong | Weak |
| Delholm et al. ,2022 | Psychology-based | Weak | Strong | Weak | Moderate | Strong | Strong | Weak |
| Ghodsbin et al.,2015 | Psychology-based | Moderate | Strong | Weak | Weak | Strong | Strong | Weak |
| Göks and Duru,2021 | Psychology-based | Weak | Strong | Strong | Weak | Strong | Strong | Weak |
| Gomez-Soria et al.,2020 | Psychology-based | Moderate | Strong | Weak | Weak | Strong | Moderate | Weak |
| Gomez-Soria et al.,2023 | Psychology-based | Moderate | Strong | Weak | Weak | Strong | Moderate | Weak |
| van der Weele et al.,2012 | Psychology-based | Moderate | Strong | Weak | Weak | Strong | Strong | Weak |
| C. X. Wang et al., 2023 | Psychology-based | Strong | Strong | Strong | Weak | Strong | Weak | Weak |
| Scogin et al.,2014 | Psychology-based | Weak | Strong | Weak | Moderate | Strong | Moderate | Weak |
| Scott et al., 2024 | Psychology-based | Weak | Strong | Weak | Moderate | Strong | Weak | Weak |
| Liao et al.,2018 | Mind-Body-Based | Strong | Strong | Strong | Moderate | Strong | Moderate | Strong |
| Tabei et al., 2024 | Mind-Body-Based | Moderate | Strong | Strong | Moderate | Strong | Strong | Strong |
| Yujie Ge et al.,2021 | Mind-Body-Based | Moderate | Strong | Strong | Moderate | Strong | Strong | Strong |
| de Lima et al.,2021 | Mind-Body-Based | Moderate | Strong | Weak | Moderate | Strong | Moderate | Moderate |
| Lee,2023 | Mind-Body-Based | Weak | Strong | Strong | Moderate | Strong | Strong | Moderate |
| Moret et al.,2022 | Mind-Body-Based | Weak | Strong | Strong | Moderate | Strong | Strong | Moderate |
| Prakhinkit et al.,2014 | Mind-Body-Based | Moderate | Strong | Weak | Moderate | Strong | Strong | Moderate |
| Shahidi et al.,2011 | Mind-Body-Based | Moderate | Strong | Weak | Moderate | Strong | Strong | Moderate |
| Makizako et al.,2020 | Mind-Body-Based | Weak | Strong | Strong | Weak | Strong | Moderate | Weak |
| Solianik et al.,2021 | Mind-Body-Based | Weak | Strong | Weak | Moderate | Strong | Strong | Weak |
| Cavalcante et al.,2021 | Mind-Body-Based | Weak | Strong | Weak | Weak | Strong | Strong | Weak |
| Brenes et al.,2007 | Mind-Body-Based | Weak | Strong | Weak | Moderate | Strong | Strong | Weak |
| Legrand and Mille,2009 | Mind-Body-Based | Weak | Strong | Weak | Moderate | Strong | Strong | Weak |
| Alegria et al.,2019 | Multicomponent | Weak | Strong | Strong | Moderate | Strong | Moderate | Moderate |
| Bae et al.,2019 | Multicomponent | Weak | Strong | Strong | Strong | Strong | Moderate | Moderate |
| Hardman et al.,2020 | Multicomponent | Moderate | Strong | Strong | Weak | Strong | Moderate | Moderate |
| Pynnonen et al.,2018 | Multicomponent | Moderate | Strong | Weak | Moderate | Strong | Strong | Moderate |
| Scazufca et al.,2022 | Multicomponent | Moderate | Strong | Weak | Moderate | Strong | Weak | Weak |
| Bøen et al.,2012 | Multicomponent | Weak | Strong | Weak | Moderate | Strong | Weak | Weak |
| Hae-Jin Ko et al.,2016 | Other | Moderate | Strong | Strong | Moderate | Strong | Moderate | Strong |
| W. L. Wang et al.,2023 | Other | Moderate | Strong | Strong | Moderate | Strong | Strong | Strong |
| Hong and Lee, 2023 | Other | Weak | Strong | Strong | Moderate | Strong | Strong | Moderate |
| Kawakami et al.,2018 | Other | Weak | Strong | Strong | Moderate | Strong | Strong | Moderate |
| Ishihara et al.,2018 | Other | Weak | Strong | Strong | Weak | Strong | Strong | Weak |

**GRADE Evidence Profile — Depressive symptoms**

Population: participants in included randomized controlled trials (RCTs)
Intervention vs comparator: listed categories vs control
Outcome: Depression symptom reduction (negative SMD indicates improvement)
Effect metric: pooled between-group standardized mean differences (SMD)

| **Intervention category** | **N**  **studies** | **n** | **Effect (SMD, 95% CI)** | **I²** | **Inconsistency** | **Indirectness** | **Imprecision** | **Publication bias** | **Overall certainty** |
| --- | --- | --- | --- | --- | --- | --- | --- | --- | --- |
| **Psychology-based** | 34 | 3608 | d = -0.415  (-0.577, -0.253) | 81% | Serious | Not serious | Not serious | Not detected | Moderate  ⊕⊕⊕⊝ |
| **Mind-Body-based** | 12 | 580 | d = -0.973  (-1.461, -0.485) | 86% | Serious | Not serious | Not serious | Not detected | Moderate  ⊕⊕⊕⊝ |
| **Multicomponent** | 5 | 973 | d = -0.470  (-1.095, 0.155) | 94% | Very serious | Not serious | Serious | Not detected | Low  ⊕⊕⊝⊝ |
| **Other** | 5 | 356 | d = -0.578  (-0.890, -0.267) | 51.7% | Serious | Not serious | Not serious | Not detected | Moderate  ⊕⊕⊕⊝ |

**Footnotes:**1) No downgrading for risk of bias as moderation analyses revealed no effect
2) Inconsistency downgraded based on I² thresholds
3) No downgrading for indirectness (between-group effects)
4) Imprecision downgraded when CI includes 0 or when CI was wide with small k/n
5) Publication bias: not detected

**GRADE Evidence Profile — Anxiety Symptoms**

Population: participants in included randomized controlled trials (RCTs)
Intervention vs comparator: listed categories vs control
Outcome: Anxiety symptom reduction (negative SMD indicates improvement)
Effect metric: pooled between-group standardized mean differences (SMD)

| **Intervention category** | **N**  **studies** | **n** | **Effect (SMD, 95% CI)** | **I²** | **Inconsistency** | **Indirectness** | **Imprecision** | **Publication bias** | **Overall certainty** |
| --- | --- | --- | --- | --- | --- | --- | --- | --- | --- |
| **Psychology-based** | 19 | 1368 | d = -0.420  (-0.612, -0.227) | 74.9% | Serious | Not serious | Not serious | Not detected | Moderate  ⊕⊕⊕⊝ |
| **Mind-Body-based** | 3 | 59 | d = -0.623  (-0.993, -0.253) | ≈0% | Not serious | Not serious | Serious | Not detected | Moderate  ⊕⊕⊕⊝ |
| **Multicomponent** | 3 | 600 | d = -0.019  (-0.325, 0.288) | 72.4% | Serious | Not serious | Serious | Not detected | Low  ⊕⊕⊝⊝ |
| **Other** | 1 | 53 | d = -0.217  (-0.731, 0.298) | 56.9% | Serious | Not serious | Serious | Not detected | Moderate  ⊕⊕⊕⊝ |

**Footnotes:**1) No downgrading for risk of bias as moderation analyses revealed no effect
2) Inconsistency downgraded based on I² thresholds
3) No downgrading for indirectness (between-group effects)
4) Imprecision downgraded when CI includes 0 or when CI was wide with small k/n
5) Publication bias: not detected
