# Supplementary material for: A meta-analysis of preventive psychosocial interventions against depressive and anxiety symptoms in older adults
Source: Psychol Med. 2026 May 14;56:e151. doi: 10.1017/S0033291726104607 (PMC13200161; doi:10.1017/S0033291726104607)
Supplement: Saldivia et al. supplementary material [file S0033291726104607sup001.zip › Supplementary File 5 Publication bias.docx]

Supplementary File 5. Results of four different publication bias detection methods for four outcomes

|  |  | Anxiety  (*k* = 30) | Anxiety follow up (*k* = 19) | Depression  (*k* = 60) | Depression follow up  (*k* = 24) |
| --- | --- | --- | --- | --- | --- |
| Begg & Mazumdar | *p* value | .041 | .049 | .001 | .131 |
| Excess significance | χ^2^(1) / *p* value | 2.102 / .926 | - | 0.184 / .666 | 0.569 / .775 |
| Sterne & Egger | *p* value | .392 | .200 | <.001 | .006 |
| Trim-and-Fill | Observed *r* | -0.369 | -0.241 | -0.530 | -0.386 |
|  | Adjusted *r* | -0.369 | -0.113 | -0.530 | -0.386 |
|  | Added studies | 0 | 6 | 0 | 0 |

*Note.* All calculations were based on random effects models; *p* values for both Begg & Mazumdar’s and Egger’s tests are 1-sided.
