# Supplementary material for: A meta-analysis of preventive psychosocial interventions against depressive and anxiety symptoms in older adults
Source: Psychol Med. 2026 May 14;56:e151. doi: 10.1017/S0033291726104607 (PMC13200161; doi:10.1017/S0033291726104607)
Supplement: Saldivia et al. supplementary material [file S0033291726104607sup001.zip › Supplementary_File_14_References_included_studies.docx]

Alegría, M., Frontera, W., Cruz-Gonzalez, M., Markle, S. L., Trinh-Shevrin, C., Wang, Y., Herrera, L., Ishikawa, R. Z., Velazquez, E., Fuentes, L., Guo, Y., Pan, J., Cheung, M., Wong, J., Genatios, U., Jimenez, A., Ramos, Z., Perez, G., Wong, J. Y., Chieng, C. K., Bartels, S. J., Duan, N., & Shrout, P. E. (2019). Effectiveness of a Disability Preventive Intervention for Minority and Immigrant Elders: The Positive Minds-Strong Bodies Randomized Clinical Trial [Article]. *American Journal of Geriatric Psychiatry, 27*(12), 1299-1313. <https://doi.org/10.1016/j.jagp.2019.08.008>

Almeida, O. P., Patel, H., Kelly, R., Ford, A., Flicker, L., Robinson, S., Araya, R., Gilbody, S., & Thompson, S. (2021). Preventing depression among older people living in rural areas: A randomised controlled trial of behavioural activation in collaborative care. *International journal of geriatric psychiatry, 36*(4), 530-539. <https://doi.org/10.1002/gps.5449>

Ayudhaya, W. S. N., Pityaratstian, N., & Jiamjarasrangsi, W. (2020). Effectiveness of Behavioral Activation in Treating Thai Older Adults with Subthreshold Depression Residing in the Community. *Clinical interventions in aging, 15*, 2363-2374. <https://doi.org/10.2147/cia.S274262>

Bae, S., Lee, S., Lee, S., Jung, S., Makino, K., Harada, K., Harada, K., Shinkai, Y., Chiba, I., & Shimada, H. (2019, Feb). The effect of a multicomponent intervention to promote community activity on cognitive function in older adults with mild cognitive impairment: A randomized controlled trial. *Complementary Therapies in Medicine, 42*, 164-169. <https://doi.org/10.1016/j.ctim.2018.11.011>

Bøen, H., Dalgard, O. S., Johansen, R., & Nord, E. (2012, May 20). A randomized controlled trial of a senior centre group programme for increasing social support and preventing depression in elderly people living at home in Norway [journal article]. *BMC Geriatrics, 12*(1), 20. <https://doi.org/10.1186/1471-2318-12-20>

Brenes, G. A., Williamson, J. D., Messier, S. P., Rejeski, W. J., Pahor, M., Ip, E., & Penninx, B. (2007, Jan). Treatment of minor depression in older adults: A pilot study comparing sertraline and exercise. *Aging & Mental Health, 11*(1), 61-68. <https://doi.org/10.1080/13607860600736372>

Calatayud Sanz, E., Gómez-Cabello, A., & Gómez-Soria, I. (2021). Analysis of the effect of cognitive stimulation program in older adults with normal cognition: Randomized clinical trial [Article]. *Anales Del Sistema Sanitario De Navarra, 44*(3), 361-372. <https://doi.org/10.23938/ASSN.0961>

Casemiro, F. G., Quirino, D. M., Andreotti Diniz, M. A., Partezani Rodrigues, R. A., Iost Pavarini, S. C., & Martins Gratao, A. C. (2018). Effects of health education in the elderly with mild cognitive impairment [Journal article]. *Revista brasileira de enfermagem, 71*, 801‐810. <https://www.scielo.br/j/reben/a/DtNnD7jSVD9fkPrDSQSkB4z/?lang=en&format=pdf>

Cavalcante, B. R., Nascimento, V. Y. S., Falck, R. S., Soares, B. O., Dias, E. F., Silva, M. S., Campos, I. R. A., de Souza, M. F., & de Araujo, R. C. (2022, Apr). Effects of Resistance Exercise with Instability on Concerns about Falling and Depressive Symptoms in Cognitively Impaired Older Adults. *International Journal of Gerontology, 16*(2), 95-99. <https://doi.org/10.6890/ijge.202204_16(2).0004>

Chan, H. C. Y., & Lo, H. H. M. (2023, 2023 May). Effects of the original zentangle method on older adults with depressive symptoms a randomized waitlist-controlled trial. *Current Psychology*. <https://doi.org/10.1007/s12144-023-04536-x>

Chan, M. F., Ng, S. E., Tien, A., Ho, R. C. M., & Thayala, J. (2013, Sep). A randomised controlled study to explore the effect of life story review on depression in older Chinese in Singapore. *Health & Social Care in the Community, 21*(5), 545-553. <https://doi.org/10.1111/hsc.12043>

Cieślik, B., Juszko, K., Kiper, P., & Szczepańska-Gieracha, J. (2023, May 7). Immersive virtual reality as support for the mental health of elderly women: a randomized controlled trial. *Virtual Real*, 1-9. <https://doi.org/10.1007/s10055-023-00797-w>

de Lima, B. E., Passos, G. S., Youngstedt, S. D., Santos, L. C. B., & Santana, M. G. (2021, Oct). Effects of Xbox Kinect exercise training on sleep quality, anxiety and functional capacity in older adults. *Journal of Bodywork and Movement Therapies, 28*, 271-275. <https://doi.org/10.1016/j.jbmt.2021.07.029>

Dear, B. F., Zou, J. B., Ali, S., Lorian, C. N., Johnston, L., Sheehan, J., Staples, L. G., Gandy, M., Fogliati, V. J., Klein, B., & Titov, N. (2015, Mar). Clinical and Cost-Effectiveness of Therapist-Guided Internet-Delivered Cognitive Behavior Therapy for Older Adults With Symptoms of Anxiety: A Randomized Controlled Trial. *Behavior Therapy, 46*(2), 206-217. <https://doi.org/10.1016/j.beth.2014.09.007>

Delhom, I., Satorres, E., & Meléndez, J. C. (2022). Emotional intelligence intervention in older adults to improve adaptation and reduce negative mood [Article]. *International Psychogeriatrics, 34*(1), 79-89. <https://doi.org/10.1017/S1041610220003579>

Denkova, E., Barry, J., Zanesco, A. P., Rooks, J., Rogers, S. L., & Jha, A. P. (2024). Online mindfulness training for older adults during the COVID-19 pandemic: a randomized controlled trial using a multi-method assessment approach [Article]. *Aging and Mental Health, 28*(1), 130-141. <https://doi.org/10.1080/13607863.2023.2242301>

Eric Kam‐Pui, L., Wong, B., Chan, P. H. S., Zhang, D. D., Sun, W., Dicken Cheong‐Chun, C., Gao, T., Ho, F., Yui Kwok, T. C., & Samuel Yeung‐Shan, W. (2022, Jan 2022). Effectiveness of a mindfulness intervention for older adults to improve emotional well‐being and cognitive function in a Chinese population: A randomized waitlist‐controlled trial. *International journal of geriatric psychiatry, 37*(1). <https://doi.org/10.1002/gps.5616>

Ge, Y. J., Liu, H., Wu, Q. W., Chen, A. J., Gao, Z. P., Xing, F. M., & Liu, G. T. (2022, Dec). Effects of a short eight Tai Chi-forms for the pre-frail elderly people in senior living communities. *Physiotherapy Theory and Practice, 38*(12), 1928-1936. <https://doi.org/10.1080/09593985.2021.1926023>

Ghodsbin, F., Sharif Ahmadi, Z., Jahanbin, I., & Sharif, F. (2015). The effects of laughter therapy on general health of elderly people referring to jahandidegan community center in shiraz, iran, 2014: a randomized controlled trial [Journal article]. *International journal of community based nursing & midwifery, 3*(1), 31‐38. <https://pubmed.ncbi.nlm.nih.gov/25553332/>

Gökşin, İ., & Aşiret, G. D. (2021). The effect of progressive muscle relaxation on the adaptation of elderly women to depression and old age: a randomised clinical trial [Journal article]. *Psychogeriatrics, 21*(3), 333‐341. <https://doi.org/10.1111/psyg.12673>

Gómez-Soria, I., Ferreira, C., Oliván-Blázquez, B., Aguilar-Latorre, A., & Calatayud, E. (2023). Effects of cognitive stimulation program on cognition and mood in older adults, stratified by cognitive levels: A randomized controlled trial [Article]. *Archives of gerontology and geriatrics, 110*, Article 104984. <https://doi.org/10.1016/j.archger.2023.104984>

Gomez-Soria, I., Peralta-Marrupe, P., & Plo, F. (2020). Cognitive stimulation program in mild cognitive impairment: a randomized controlled trial [Journal article]. *Dementia & neuropsychologia, 14*(2), 110‐117. <https://doi.org/10.1590/1980-57642020dn14-020003>

Hardman, R. J., Meyer, D., Kennedy, G., Macpherson, H., Scholey, A. B., & Pipingas, A. (2020, May). Findings of a Pilot Study Investigating the Effects of Mediterranean Diet and Aerobic Exercise on Cognition in Cognitively Healthy Older People Living Independently within Aged-Care Facilities: The Lifestyle Intervention in Independent Living Aged Care (LIILAC) Study. *Current Developments in Nutrition, 4*(5), Article nzaa077. <https://doi.org/10.1093/cdn/nzaa077>

Hong, D., & Lee, S. H. (2023, Jan-Dec). Effectiveness of the non-face-to-face comprehensive elderly care application "smart silver care" for community-dwelling elderly: A randomized controlled trial. *Digital Health, 9*, 20552076231197340. <https://doi.org/10.1177/20552076231197340>

Ishihara, M., Saito, T., Sakurai, T., Shimada, H., & Arai, H. (2018). Effect of a positive photo appreciation program on depressive mood in older adults: A pilot randomized controlled trial [Article]. *International Journal of Environmental Research and Public Health, 15*(7), Article 1472. <https://doi.org/10.3390/ijerph15071472>

Jiang, D., Tang, V. F. Y., Kahlon, M., Chow, E. O. W., Yeung, D. Y. L., Aubrey, R., & Chou, K. L. (2024). Effects of Wisdom-Enhancement Narrative-Therapy and Empathy-Focused interventions on loneliness over 4 weeks among older adults: A Randomized Controlled Trial [Article]. *American Journal of Geriatric Psychiatry*. <https://doi.org/10.1016/j.jagp.2024.07.003>

Joling, K. J., van Hout, H. P., van't Veer-Tazelaar, P. J., van der Horst, H. E., Cuijpers, P., van de Ven, P. M., & van Marwijk, H. W. (2011). How effective is bibliotherapy for very old adults with subthreshold depression? A randomized controlled trial [Journal article]. *American Journal of Geriatric Psychiatry, 19*(3), 256‐265. <https://doi.org/10.1097/JGP.0b013e3181ec8859>

Kawakami, R., Sawada, S. S., Ito, T., Gando, Y., Fukushi, T., Yoshino, A., Kurita, S., Oka, K., Sakamoto, S., & Higuchi, M. (2019, Aug). Effect of watching professional baseball at a stadium on health-related outcomes among Japanese older adults: A randomized controlled trial. *Geriatrics & gerontology international, 19*(8), 717-722. <https://doi.org/10.1111/ggi.13687>

Klainin-Yobas, P., Kowitlawakul, Y., Lopez, V., Tang, C. T., Hoek, K. E., Gan, G. L., Lei, F., Rawtaer, I., & Mahendran, R. (2019, Oct). The effects of mindfulness and health education programs on the emotional state and cognitive function of elderly individuals with mild cognitive impairment: A randomized controlled trial. *Journal of Clinical Neuroscience, 68*, 211-217. <https://doi.org/10.1016/j.jocn.2019.05.031>

Ko, H. J., Youn, C. H., Kim, S. H., & Kim, S. Y. (2016). Effect of Pet Insects on the Psychological Health of Community-Dwelling Elderly People: a Single-Blinded, Randomized, Controlled Trial [Journal article]. *Gerontology, 62*(2), 200‐209. <https://doi.org/10.1159/000439129>

Lai, C. K. Y., Chin, K. C. W., Zhang, Y., & Chan, E. A. (2019, Sep). Psychological outcomes of life story work for community-dwelling seniors: A randomised controlled trial. *International Journal of Older People Nursing, 14*(3), Article e12238. <https://doi.org/10.1111/opn.12238>

Lee, K. (2023, Apr 12). Home-Based Exergame Program to Improve Physical Function, Fall Efficacy, Depression and Quality of Life in Community-Dwelling Older Adults: A Randomized Controlled Trial. *Healthcare, 11*(8). <https://doi.org/10.3390/healthcare11081109>

Legrand, F. D., & Mille, C. R. (2009). The effects of 60 minutes of supervised weekly walking (in a single vs. 3-5 session format) on depressive symptoms among older women: Findings from a pilot randomized trial [Article]. *Mental Health and Physical Activity, 2*(2), 71-75. <https://doi.org/10.1016/j.mhpa.2009.09.002>

Liao, S. J., Chong, M. C., Tan, M. P., & Chua, Y. P. (2019, Mar-Apr). Tai Chi with music improves quality of life among community-dwelling older persons with mild to moderate depressive symptoms: A cluster randomized controlled trial. *Geriatric Nursing, 40*(2), 154-159. <https://doi.org/10.1016/j.gerinurse.2018.08.001>

Lwi, S. J., Paulraj, S. R., Schendel, K., Dempsey, D. G., Curran, B. C., Herron, T. J., & Baldo, J. V. (2023, May). A Randomized, Controlled Pilot Study of Mindfulness-Based Stress Reduction in Healthy Older Adults. *Clinical Gerontologist, 46*(3), 330-345. <https://doi.org/10.1080/07317115.2022.2137075>

Makizako, H., Tsutsumimoto, K., Doi, T., Makino, K., Nakakubo, S., Liu-Ambrose, T., & Shimada, H. (2020). Exercise and horticultural programs for older adults with depressive symptoms and memory problems: A randomized controlled trial [Article]. *Journal of Clinical Medicine, 9*(1), Article 99. <https://doi.org/10.3390/jcm9010099>

Marchant, N. L., Barnhofer, T., Coueron, R., Wirth, M., Lutz, A., Arenaza-Urquijo, E. M., Collette, F., Poisnel, G., Demnitz-King, H., Schild, A. K., Coll-Padros, N., Delphin-Combe, F., Whitfield, T., Schlosser, M., Gonneaud, J., Asselineau, J., Walker, Z., Krolak-Salmon, P., Molinuevo, J. L., Frison, E., Chételat, G., Jessen, F., & Klimecki, O. M. (2021). Effects of a Mindfulness-Based Intervention versus Health Self-Management on Subclinical Anxiety in Older Adults with Subjective Cognitive Decline: The SCD-Well Randomized Superiority Trial [Article]. *Psychotherapy and Psychosomatics, 90*(5), 341-350. <https://doi.org/10.1159/000515669>

Moret, B., Nucci, M., & Campana, G. (2022, Nov). Effects of exergames on mood and cognition in healthy older adults: A randomized pilot study. *Frontiers in Psychology, 13*, Article 1018601. <https://doi.org/10.3389/fpsyg.2022.1018601>

Prakhinkit, S., Suppapitiporn, S., Tanaka, H., & Suksom, D. (2014, May). Effects of Buddhism Walking Meditation on Depression, Functional Fitness, and Endothelium-Dependent Vasodilation in Depressed Elderly. *Journal of Alternative and Complementary Medicine, 20*(5), 411-416. <https://doi.org/10.1089/acm.2013.0205>

Pynnonen, K., Tormakangas, T., Rantanen, T., Tiikkainen, P., & Kallinen, M. (2018). Effect of a social intervention of choice vs. control on depressive symptoms, melancholy, feeling of loneliness, and perceived togetherness in older Finnish people: a randomized controlled trial. *Aging & Mental Health, 22*(1), 77-84. <https://doi.org/10.1080/13607863.2016.1232367>

Scazufca, M., Nakamura, C. A., Seward, N., Moreno-Agostino, D., van de Ven, P., Hollingworth, W., Peters, T. J., & Araya, R. (2022). A task-shared, collaborative care psychosocial intervention for improving depressive symptomatology among older adults in a socioeconomically deprived area of Brazil (PROACTIVE): a pragmatic, two-arm, parallel-group, cluster-randomised controlled trial [Article]. *The lancet healthy longevity, 3*(10), e690-e702. <https://doi.org/10.1016/S2666-7568(22)00194-5>

Scogin, F. R., Moss, K., Harris, G. M., & Presnell, A. H. (2014, Mar). Treatment of depressive symptoms in diverse, rural, and vulnerable older adults. *International journal of geriatric psychiatry, 29*(3), 310-316. <https://doi.org/10.1002/gps.4009>

Scott, J. E. T., Mazzucchelli, T. G., Walker, R., Luszcz, M. A., & Windsor, T. D. (2024, Aug 26). A randomized controlled trial of a behavioral activation intervention to increase engagement with life and wellbeing in older adults. *Gerontology*. <https://doi.org/10.1159/000541079>

Shahidi, M., Mojtahed, A., Modabbernia, A., Mojtahed, M., Shafiabady, A., Delavar, A., & Honari, H. (2011, Mar). Laughter Yoga versus group exercise program in elderly depressed women: a randomized controlled trial. *International journal of geriatric psychiatry, 26*(3), 322-327. <https://doi.org/10.1002/gps.2545>

Shih, V. W. Y., Chan, W. C., Tai, O. K., Wong, H. L., Cheng, C. P. W., & Wong, C. S. M. (2021, Jun 2021). Mindfulness-Based Cognitive Therapy for Late-Life Depression: a Randomised Controlled Trial. *East asian archives of psychiatry, 31*(2), 27. <https://doi.org/10.12809/eaap2075>

Solianik, R., Mickeviciene, D., Zlibinaite, L., & Cekanauskaite, A. (2021, Jul). Tai chi improves psychoemotional state, cognition, and motor learning in older adults during the COVID-19 pandemic. *Experimental Gerontology, 150*, Article 111363. <https://doi.org/10.1016/j.exger.2021.111363>

Srisuwan, P., Nakawiro, D., Chansirikarnjana, S., Kuha, O., Chaikongthong, P., & Suwannagoot, T. (2020). Effects of a Group-Based 8-Week Multicomponent Cognitive Training on Cognition, Mood and Activities of Daily Living among Healthy Older Adults: A One-Year Follow-Up of a Randomized Controlled Trial [Article]. *Journal of prevention of alzheimer's disease, 7*(2), 112-121. <https://doi.org/10.14283/jpad.2019.42>

Szczepańska-Gieracha, J., Cieślik, B., Serweta, A., & Klajs, K. (2021, May 1). Virtual Therapeutic Garden: A Promising Method Supporting the Treatment of Depressive Symptoms in Late-Life: A Randomized Pilot Study. *J Clin Med, 10*(9). <https://doi.org/10.3390/jcm10091942>

Tabei, M., Ravari, A., Kataria, M., Mirzaei, T., & Kamiab, Z. (2024, Aug 6). The effect of laughter yoga and music intervention on depression, anxiety, and stress in the Rafsanjan-Iran aged: a randomized clinical trial study. *Aging & Mental Health*, 1-8. <https://doi.org/10.1080/13607863.2024.2385454>

Tanaka, M., Kusaga, M., Nyamathi, A. M., & Tanaka, K. (2019, Feb). Effects of Brief Cognitive Behavioral Therapy for Insomnia on Improving Depression Among Community-Dwelling Older Adults: A Randomized Controlled Comparative Study. *Worldviews on Evidence-Based Nursing, 16*(1), 78-86. <https://doi.org/10.1111/wvn.12342>

Titov, N., Dear, B. F., Ali, S., Zou, J. B., Lorian, C. N., Johnston, L., Terides, M. D., Kayrouz, R., Klein, B., Gandy, M., & Fogliati, V. J. (2015, Mar). Clinical and Cost-Effectiveness of Therapist-Guided Internet-Delivered Cognitive Behavior Therapy for Older Adults With Symptoms of Depression: A Randomized Controlled Trial. *Behavior Therapy, 46*(2), 193-205. <https://doi.org/10.1016/j.beth.2014.09.008>

Titov, N., Fogliati, V. J., Staples, L. G., Gandy, M., Johnston, L., Wootton, B., Nielssen, O., & Dear, B. F. (2016, Jan 2016). Treating anxiety and depression in older adults: randomised controlled trial comparing guided V. self-guided internet-delivered cognitive–behavioural therapy. *BJPsych Open, 2*(1), 50-58. <https://doi.org/10.1192/bjpo.bp.115.002139>

Tran, T., Finlayson, M., Nalder, E., Trothen, T., & Donnelly, C. (2023). Occupational Therapist-Led Mindfulness Training Program for Older Adults Living with Early Cognitive Decline in Primary Care: A Pilot Randomized Controlled Trial [Article]. *Journal of Alzheimer's Disease Reports, 7*(1), 775-790. <https://doi.org/10.3233/ADR-230006>

van der Weele, G. M., de Waal, M. W. M., van den Hout, W. B., de Craen, A. J. M., Spinhoven, P., Stijnen, T., Assendelft, W. J. J., van der Mast, R. C., & Gussekloo, J. (2012, Jul). Effects of a stepped-care intervention programme among older subjects who screened positive for depressive symptoms in general practice: the PROMODE randomised controlled trial. *Age and ageing, 41*(4), 482-488. <https://doi.org/10.1093/ageing/afs027>

Wahbeh, H., Goodrich, E., & Oken, B. S. (2016, Mar-Apr). Internet-based Mindfulness Meditation for Cognition and Mood in Older Adults: A Pilot Study. *Alternative Therapies in Health and Medicine, 22*(2), 44-53. <https://www.ncbi.nlm.nih.gov/pmc/articles/PMC4874472/pdf/nihms784909.pdf>

Wang, C. X., Wang, C. Y., Wang, J. F., Yu, N. X., Tang, Y., Liu, Z. K., & Chen, T. Y. (2023, Jul). Effectiveness of Solution-Focused Group Counseling on Depression and Cognition Among Chinese Older Adults: A Cluster Randomized Controlled Trial. *Research on Social Work Practice, 33*(5), 530-543. <https://doi.org/10.1177/10497315221119991>

Wang, W. L., Lee, K. T., Lin, W. C., Yang, Y. C., & Tsai, C. L. (2023). The effects of a magic-based intervention on self-esteem, depressive symptoms, and quality of life among community-dwelling older adults: a randomised controlled trial [Article]. *Psychogeriatrics, 23*(4), 701-712. <https://doi.org/10.1111/psyg.12987>

Xie, J., He, G., Ding, S., Pan, C., Zhang, X., Zhou, J., & Iennaco, J. D. (2019). A randomized study on the effect of modified behavioral activation treatment for depressive symptoms in rural left-behind elderly [Journal article]. *Psychotherapy Research, 29*(3), 372‐382. <https://doi.org/10.1080/10503307.2017.1364444>
