# Supplementary figures and images for: A meta-analysis of preventive psychosocial interventions against depressive and anxiety symptoms in older adults
Source: Psychol Med. 2026 May 14;56:e151. doi: 10.1017/S0033291726104607 (PMC13200161; doi:10.1017/S0033291726104607)

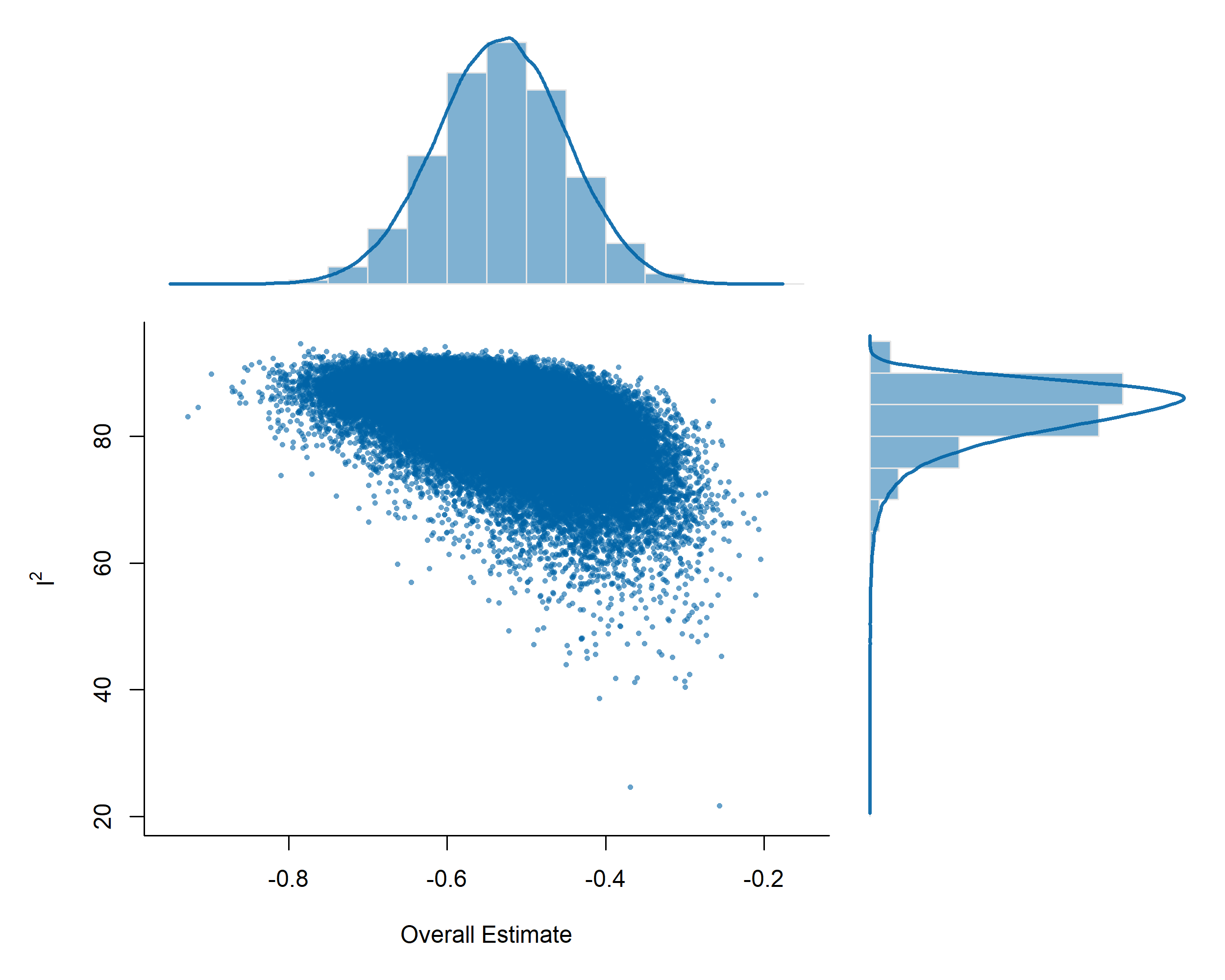

Supplement: Saldivia et al. supplementary material [file S0033291726104607sup001.zip › Supplementary File 9 gosh_depression.png]

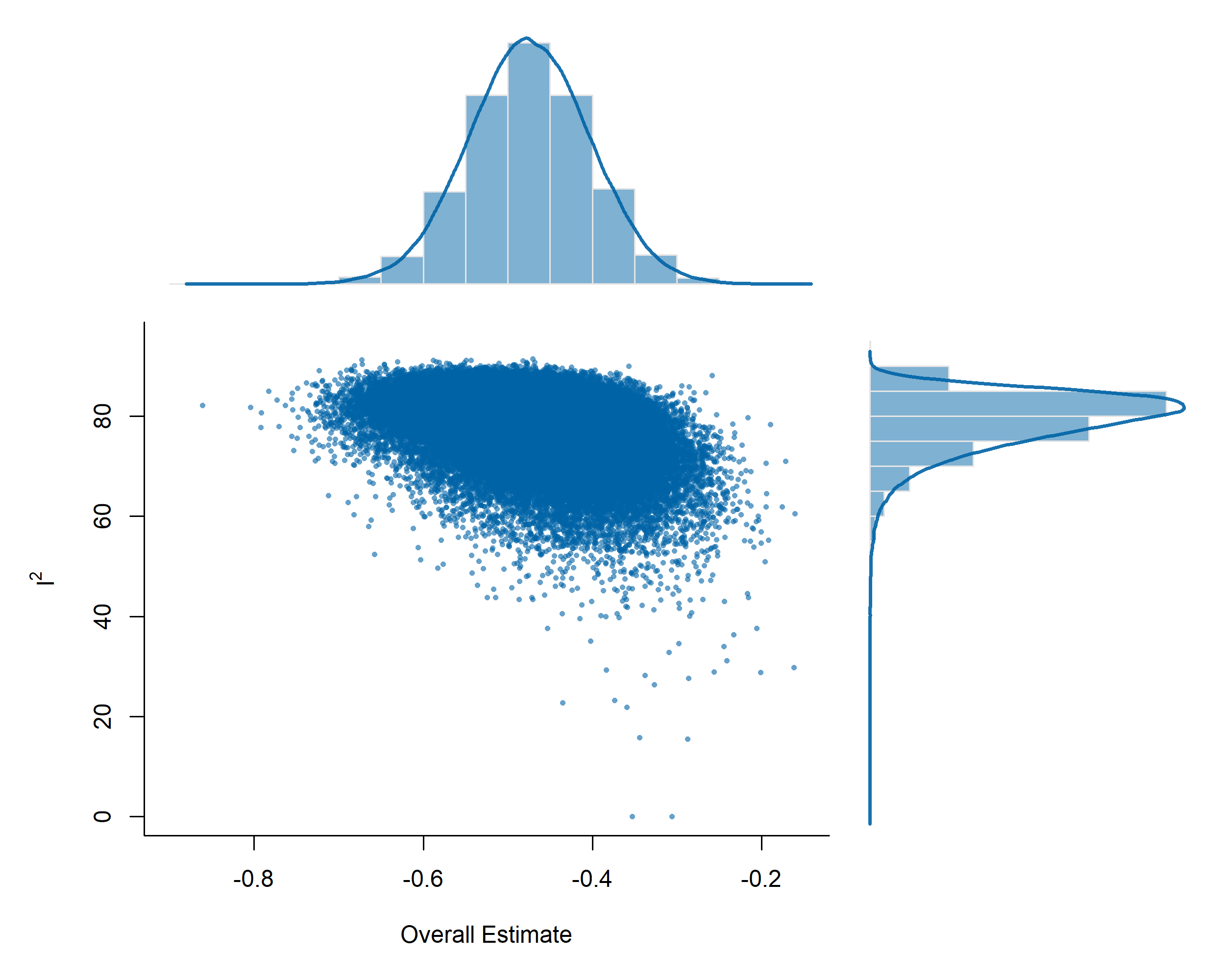

Supplement: Saldivia et al. supplementary material [file S0033291726104607sup001.zip › Supplementary File 10 gosh_depressionwo.png]

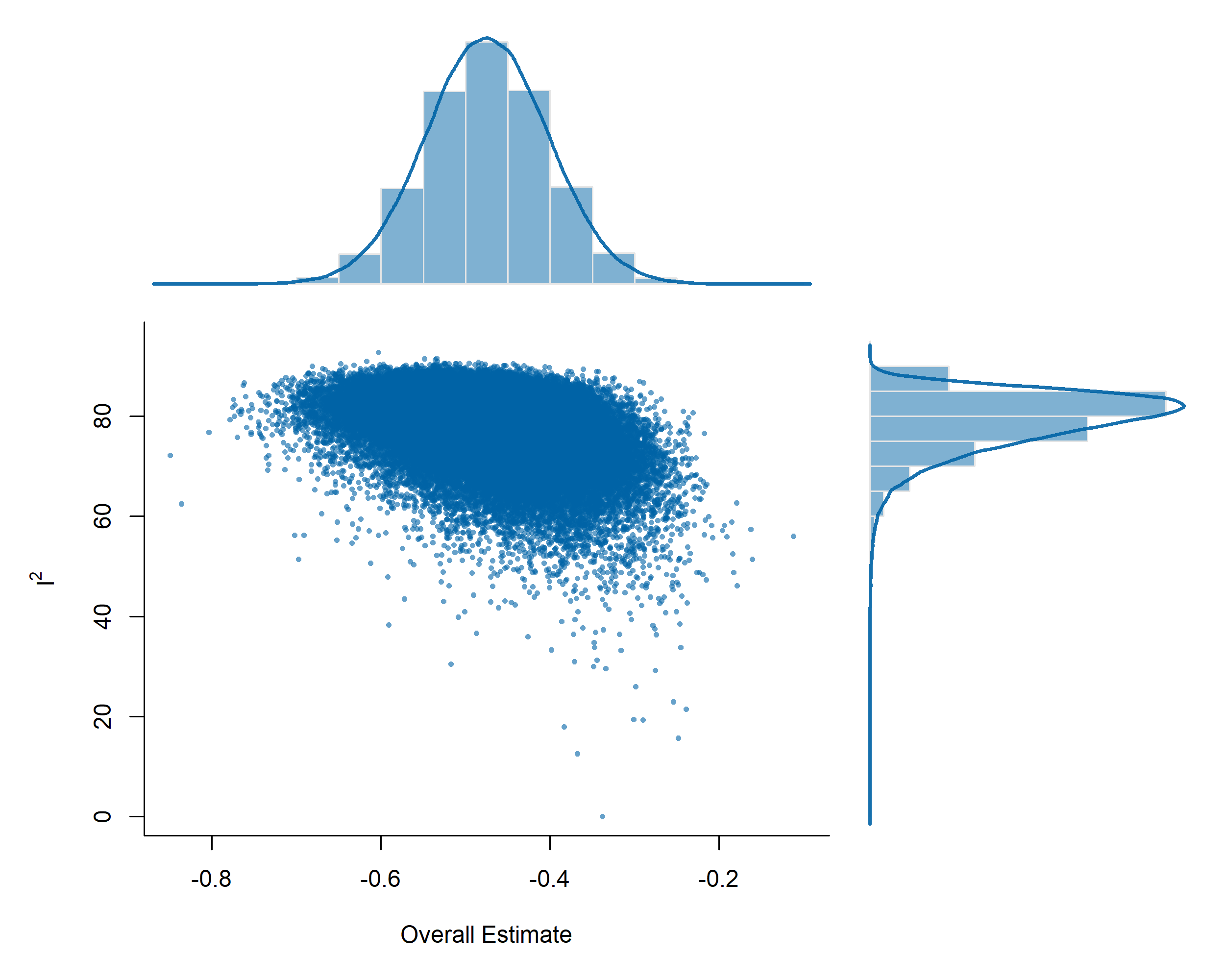

Supplement: Saldivia et al. supplementary material [file S0033291726104607sup001.zip › Supplementary File 11 gosh_depressionfu.png]

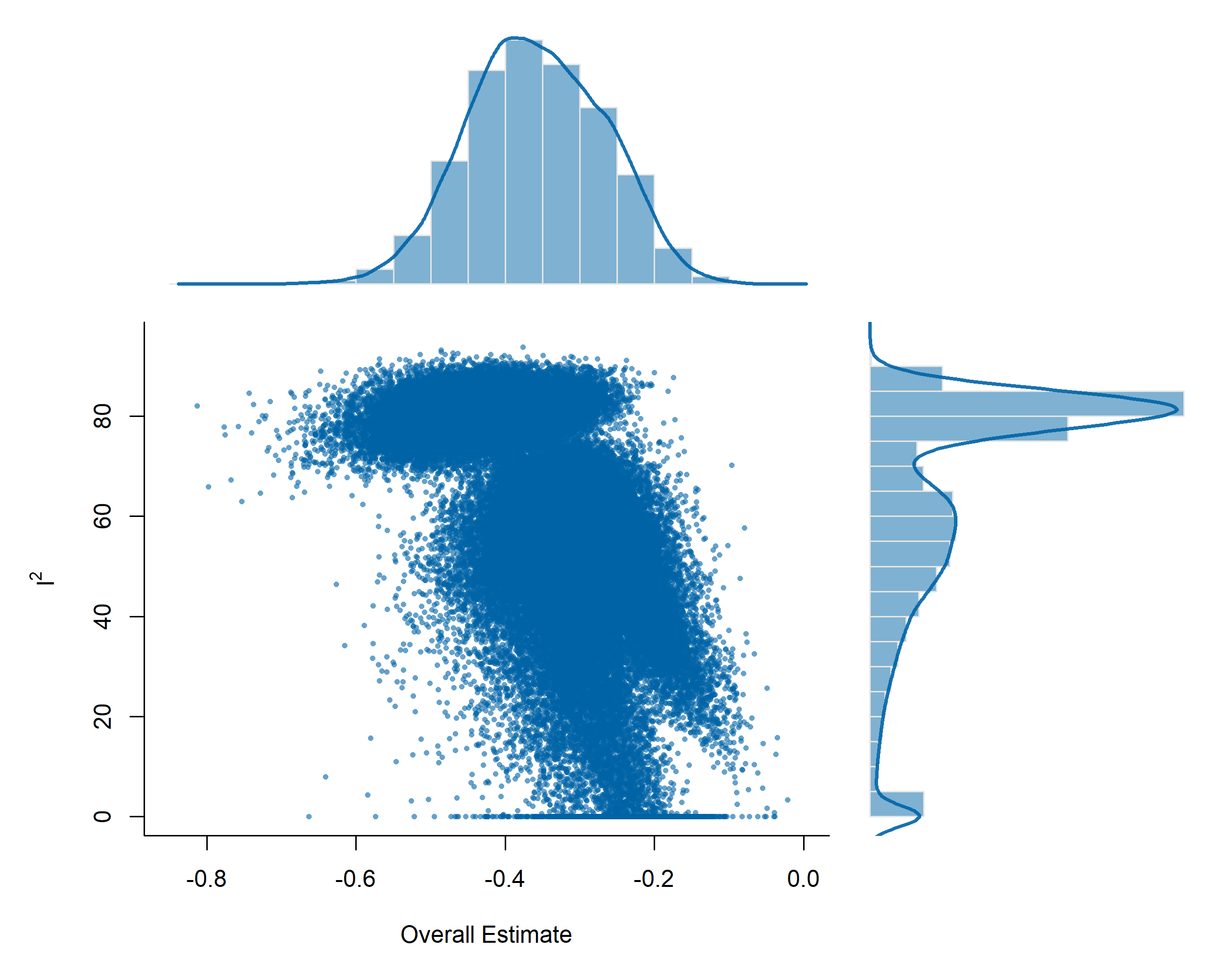

Supplement: Saldivia et al. supplementary material [file S0033291726104607sup001.zip › Supplementary File 12 gosh_anxiety.png]

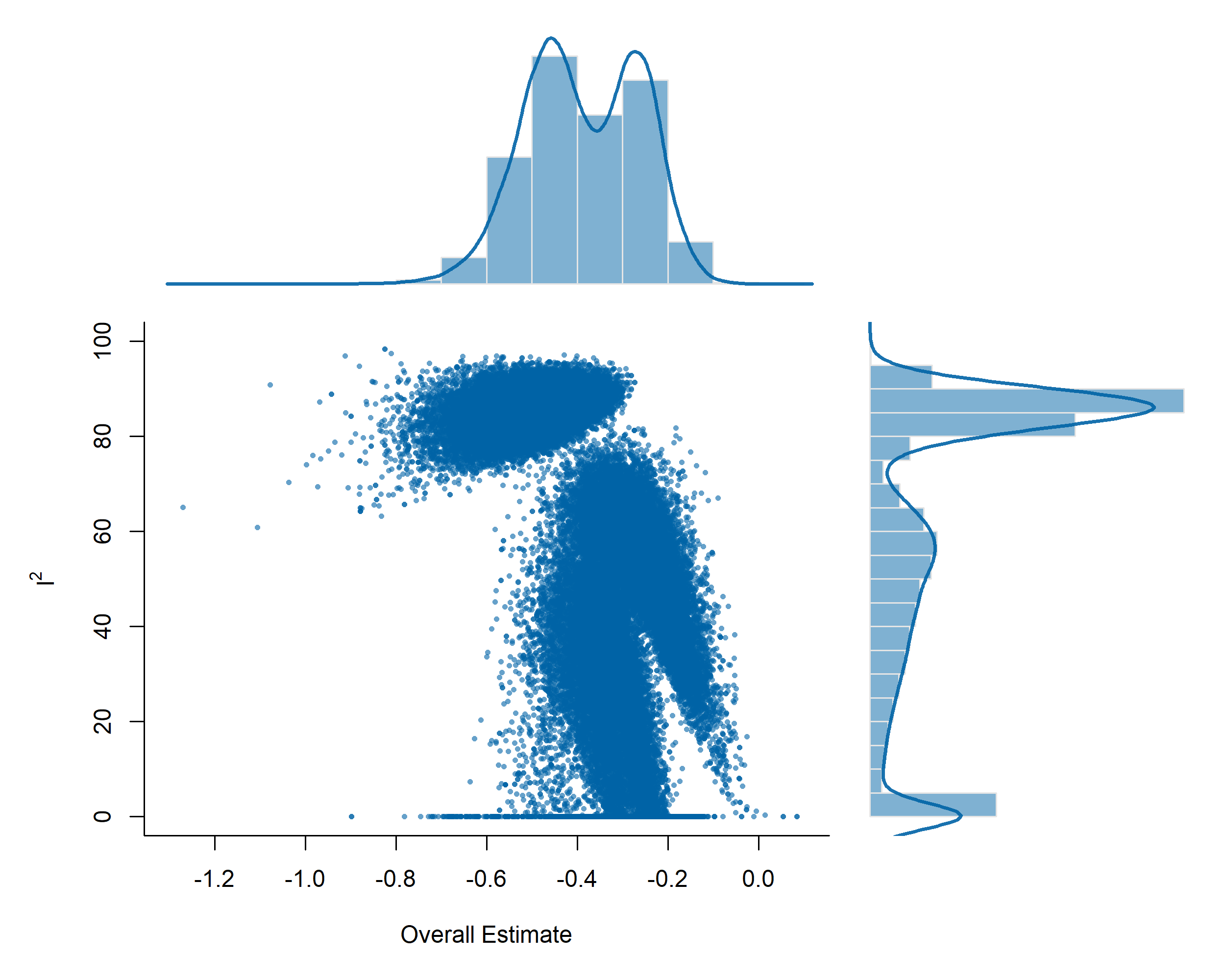

Supplement: Saldivia et al. supplementary material [file S0033291726104607sup001.zip › Supplementary File 13 gosh_anxietyfu.png]
